# Association between intrinsic capacity and trajectories of physical function among older adults: findings from a 10-year longitudinal cohort study

**DOI:** 10.3389/fpubh.2026.1893548

**Published:** 2026-09-23

**Authors:** Pengqun Leng, Ying Lei, Li Wang, Shan He, Qiping An, Xinyu Wang, Hongmei Lu, Wenqi He, Ling Luo, Dan Liu, Wen Zeng

**Affiliations:** 1 Nursing School, Guizhou University of Traditional Chinese Medicine, Guiyang, China; 2 Guizhou Provincial People’s Hospital, Guiyang, China

**Keywords:** activities of daily living, healthy aging, intrinsic capacity, physical function, trajectories

## Abstract

**Objectives:**

To identify trajectories of basic activities of daily living (BADL) and instrumental activities of daily living (IADL) and determine whether the baseline intrinsic capacity (IC) deficit score predicts 10-year functional decline in older adults.

**Methods:**

Data were from the Chinese Longitudinal Healthy Longevity Survey (2008–2018, *N* = 2,045). A six-domain IC deficit score was constructed using the Integrated Care for Older People (ICOPE) framework. Group-based trajectory modeling, generalized linear models, and multilevel mixed-effects models (stratified by age) were used.

**Results:**

Two BADL trajectories (high-stable 81.9%; high-declining 18.1%) and three IADL trajectories (mild, moderate, severe decline) were identified. A higher IC deficit score predicted declining BADL (odds ratio [OR] = 1.22, 95% confidence interval [CI]: 1.09–1.37), severe IADL decline (OR = 1.94, 95%CI: 1.66–2.27), and moderate IADL decline (OR = 1.37, 95%CI: 1.22–1.53). Each one-unit increase in the IC deficit score was associated with an additional annual BADL decline of 0.007 points (β = −0.007, 95% CI: −0.010 to −0.003, *p* = 0.001), equivalent to approximately 0.07 points over 10 years on the 0–6 scale. This effect was statistically significant but small in clinical magnitude and is better interpreted as a marker of preclinical functional change than as overt loss of independence. The IC × time association for IADL was not statistically significant in the primary complete-case analysis and was sensitive to missing-data assumptions. Annual IADL decline was greater for adults aged ≥80 vs. 60–79.

**Conclusion:**

A higher IC deficit score was associated with adverse physical-function trajectories and a small acceleration in BADL decline. Although the annual effect was unlikely to represent clinically perceptible loss of independence, it may indicate early functional vulnerability.

## 1 Introduction

Over the past decades, increased life expectancy and decreased mortality associated with socioeconomic and medical development have rapidly expanded aging populations (1). The global estimates of older adults will reach 1.5 billion in 2050, accounting for 16.0% of the population (2). In the context of rapid population aging, the World Health Organization (WHO) clearly outlined a novel framework of healthy aging based on individuals’ functional abilities rather than disease-based conceptualizations in 2015 (3).

A key challenge for healthy aging arises from maintaining physical function (3, 4), given the high prevalence of age-related functional declines or disabilities among older adults (5, 6). Physical function is usually characterized by the ability to perform daily activities independently (7), including Basic Activities of Daily Living (BADL) and Instrumental Activities of Daily Living (IADL) (3). Particularly, BADL is an essential measurement for assessing the self-care ability of individuals for fundamental daily tasks (8), and IADL reflects a more complex but essential ability of individuals to engage in social interactions (9). Physical function is a dynamic process that changes with aging (3). Thus, it is critical to track the long-term trajectories of physical function over time, and early detection of declining physical function trajectories might be beneficial for informing effective strategies to improve physical function for healthy aging (7). However, to our knowledge, most studies related to physical function are cross-sectional. A few studies have examined the trajectories of physical function in older adults. Still, they have focused only on a narrow population (e.g., a younger group of older adults (7)) or frail older adults (10). For instance, Jonkman et al. (7) investigated trajectories of physical function measured by a combination of 2 items on BADL and four items on IADL in community-dwelling Italian and Dutch older adults aged 60–70 years. They identified three distinct trajectories of physical function: no/little decline, intermediate decline, and severe decline. Different from the study of Jonkman et al., Mueller-Schotte et al. (10) examined trajectories of IADL in community-living frail older adults aged 60 and over, and they found a declined IADL trajectory with age. Additionally, older adults with vision, hearing, or dual sensory loss were associated with higher levels of IADL limitations. Because of the narrow target population, those studies limit the generalizability to all older adults. Indeed, some studies have estimated the long-term trajectories of physical function in general older adults in late life and found heterogeneous functional trajectories, and factors such as age, sex, educational level, initial cognitive function, initial disability, medication use, self-reported health status were associated with those trajectories heterogeneity (11, 12). However, few studies estimate the association between intrinsic capacity (IC) and physical function trajectories.

IC is defined as “the composite of all the physical and mental capacities that an individual can draw on” (p. 28) (3), including cognitive capacity, psychological capacity, locomotor capacity, vitality, visual capacity, and hearing capacity according to the guidelines for Integrated Care for Older People (ICOPE) (13). In WHO reports on healthy aging (3, 13), IC is recognized as a promising multidimensional assessment to comprehensively capture the reserves of older adults, especially for the most vulnerable individuals (14). Prior research identified four distinct IC trajectories (i.e., high-stable, normal-stable, sensory-dysfunction, and all-dysfunction class) and suggested that individuals with poor IC trajectory patterns were associated with worse ADL scores (15). However, little is known about the predictive value of IC on distinct long-term trajectories of physical function. To fill this knowledge gap, the current study aimed to (1) identify the distinct trajectories of BADL and IADL over 10 years among older adults in China using a nationally representative cohort study and (2) investigate the association between IC and long-term trajectories of BADL and IADL.

## 2 Materials and methods

### 2.1 Data sources and study population

The current study sample was obtained from a nationally representative cohort study, the Chinese Longitudinal Healthy Longevity Survey (CLHLS) (16), which began recruitment in 1998 and covered the population of 23 provinces in China by using a multistage random-sampling method for a disproportionate target (17). We used the 2008–2018 longitudinal dataset from the CLHLS, where respondents were interviewed initially in 2008 (wave 1) and subsequently followed up in 2011 (wave 2), 2014 (wave 3), and 2018 (wave 4). Participants were eligible for the complete-case analysis if they were aged 60 years or older in 2008, had complete baseline data on BADL, IADL, the IC deficit score, and covariates, and had complete BADL and IADL measurements at all four waves (2008, 2011, 2014, and 2018). Of the 14,658 eligible participants at baseline, 2,045 (14.0%) met these complete-case criteria and were included in the primary trajectory analysis. (see Supplementary e-Flowchart; Supplementary Table S1).

### 2.2 Measures

#### 2.2.1 Physical function

Physical function was evaluated by BADL and IADL in the CLHLS (2008–2018) (18) at each wave. BADL was measured through six items: bathing, dressing, toilet, indoor transfer, continence, and eating (19, 20). Each item was coded as 1 (“completely independent”) or 0 (“partially dependent or completely dependent”), resulting in a BADL score ranging from 0 to 6. A higher BADL score indicates a better level of functional independence in performing BADL (19). The BADL items in CLHLS correspond closely to the six domains of the Katz Index of Independence in Activities of Daily Living.

IADL was measured based on eight items, including the ability to (1) visit neighbors, (2) go shopping, (3) cook a meal, (4) wash clothing, (5) walk continuously for 1 kilometer, (6) lift a weight of 5 kg, (7) crouch and stand up three times, and (8) use public transportation (20). This modified IADL measure combines traditional instrumental tasks (visiting neighbors, shopping, cooking, washing, using public transportation) with physical performance tasks (walking 1 km, lifting 5 kg, crouching/standing), which enhances its sensitivity to early functional decline but distinguishes it from purely instrumental scales such as the Lawton-Brody IADL instrument. Each item was coded as 1 (“can complete the task independently”) or 0 (“needs some help or cannot complete the task”). IADL scores range from 0 to 8, with higher scores indicating a better level of IADL function (18). Physical function measured by BADL/IADL at different waves is displayed in Supplementary Table S2.

#### 2.2.2 Intrinsic capacity

Based on the WHO ICOPE guidelines (13), IC is characterized by six key domains: (1) cognitive capacity, (2) psychological capacity, (3) locomotor capacity, (4) vitality, (5) visual capacity, and (6) hearing capacity. Each domain was coded as 1 (“Impaired”) or 0 (“Not impaired”) (shown in Supplementary Table S3). The total IC deficit score ranges from 0 to 6, with higher scores indicating a greater number of impaired domains (i.e., more severe decline in intrinsic capacity). This terminology is used consistently throughout the manuscript; a high score should always be interpreted as worse intrinsic capacity (13).

Cognitive capacity was assessed using the Chinese version of the Mini-Mental State Examination (MMSE; range, 0–30). In the primary IC deficit score, cognitive impairment was operationally defined as an MMSE score <24, consistent with previous CLHLS research (21, 22). Because MMSE performance and appropriate screening cut-offs are influenced by educational attainment, particularly among individuals with little or no formal education, this threshold was treated as an operational screening definition rather than an education-invariant diagnostic criterion (23). Psychological capacity was measured by two questions: (1) “Do you often feel fearful or anxious?” and (2) “Do you often feel lonely and isolated?” Psychological capacity was coded as 1 if either question was answered as “always” “often,” “sometimes,” or “seldom,” or as 0 if both answers were “never.”

Locomotor capacity was assessed using three physical performance tests from the CLHLS questionnaire: picking up a book from the floor, standing up from a chair, and turning approximately 360°. Locomotor capacity was coded as impaired (1) if participants could not perform any of the three tests, or not impaired (0) if they performed all tests.

Based on the WHO ICOPE framework and previous IC research (24), vitality was assessed using body mass index (BMI), calculated as weight in kilograms divided by height in meters squared. In our operational coding, vitality was classified as impaired when BMI was <18.5 or ≥25.0 kg/m^2^ and as not impaired when BMI was 18.5–24.9 kg/m^2^ (Supplementary Table S3).

The test measured the visual domain: “Can the interviewee see a break in the circle on the cardboard sheet when lit by a flashlight and distinguish where the break is located?” Participants were coded as 1 if they responded “can see only, cannot distinguish,” “cannot see,” or “blind,” as 0 if they responded “can see and distinguish.”

Hearing capacity was assessed by asking, “Was the interviewee able to hear what you said?” Hearing capacity was coded as 1 if participants could not hear, needed a hearing aid, or partly heard even with a hearing aid, or 0 if they could hear without a hearing aid.

#### 2.2.3 Potential confounders

Potential confounders were collected at baseline (in 2008). Sociodemographic characteristics included age (60–79 years old, ≥80 years old), sex (male, female), ethnicity (Han, minority), educational level (literacy, illiteracy), marital status (married/separated, divorced/widowed/never married), and self-rated economic status (rich, fair, poor). Lifestyle factors included smoking status (never, ever/present), alcohol consumption status (never, ever/present), and physical activity/exercise status (never, ever/present). Health-related factors included self-rated health (good, fair, bad) and number of chronic diseases (0, 1, ≥2) at baseline (2008) (shown in the Supplementary Table S4).

### 2.3 Statistical analysis

The primary trajectory and regression analyses were conducted in the complete-case survivor cohort. Participants with incomplete BADL or IADL data at any follow-up wave were excluded from the primary analysis. To further assess potential selection into the primary complete-case cohort, baseline characteristics were compared between participants included in the primary analysis and baseline-eligible participants who were not included because of incomplete follow-up. Continuous variables were compared using Welch’s two-sample *t* test, and categorical variables were compared using Pearson’s chi-square test.

#### 2.3.1 Identifying trajectories of physical function

Stata 15.1 (StataCorp LLC) was used to perform all the analyses. The group-based trajectory modeling (GBTM) approach developed by Nagin and colleagues (25, 26) was used to identify the long-term trajectories of physical function (BADL and IADL) in older adults. We fitted models with one to four trajectory groups for both outcomes. The optimal clusters of trajectories were chosen based on the following criteria: (1) the Bayesian information criterion (BIC): clusters of trajectories with BIC approaching 0 were selected; (2) the average posterior probabilities (AvePP) higher than 0.7 for all groups; and (3) the odds of correct classification (OCC) greater than 5 (26). For BADL, although the three-group model showed a slightly better BIC, one group contained fewer than 5% of the sample and had an AvePP below 0.7, indicating unreliable classification. The two-group model was therefore selected because all groups met the AvePP and OCC thresholds and the trajectory shapes were clinically interpretable. For IADL, the two-group model failed to capture the heterogeneous decline patterns evident in the data, while the four-group model produced a group with an unstable trajectory and low classification quality. The three-group model provided the best balance of statistical fit, classification adequacy, and clinical meaningfulness. Detailed model fit statistics for all candidate models are presented in Supplementary Tables S6, S8. After distinct trajectory groups were identified, each group was named according to its baseline level and rate of change over time.

#### 2.3.2 The associations between IC and physical function trajectories

After identifying the trajectory groups of BADL and IADL, we used the generalized linear model with binomial family and default logit link and multinomial logistic regression models to examine the associations of IC with BADL and IADL trajectories, respectively.

#### 2.3.3 The associations between IC and the annual rates of change in physical function

We used a multilevel mixed-effects linear model (MMELM) for repeated measures to evaluate longitudinal associations of IC with the annual rates of change in BADL and IADL during follow-up because both BADL and IADL had repeated measurements. Models for BADL and IADL included terms for IC at baseline (the primary explanatory variable), time (years from the baseline to the end of the follow-up), and their interaction. The slope of the time trajectory indicated the annual rates of change in BADL and IADL, and the interactions of IC and time indicated the associations of IC and the annual rates of change in BADL and IADL. Both unadjusted and adjusted models were assessed. In the models, the slopes and intercepts of longitudinal changes in BADL and IADL were allowed to vary for each individual as random effects. Statistical significance was indicated by a 2-sided *p* < 0.05. Model assumptions for the multilevel mixed-effects linear model were verified by inspecting residual plots for normality and homoscedasticity, and by visually checking the random effects for approximate normality. The generalized linear models were examined using standard diagnostic plots. All models met the required assumptions satisfactorily.

#### 2.3.4 Stratified analysis

To lower the potential confounding effect of age on the study results, we performed stratified analysis by age group to identify BADL and IADL trajectories and to examine the association between IC and BADL and IADL trajectories within each age group.

#### 2.3.5 Sensitivity analyses for missing follow-up data

Given the substantial loss to follow-up, we conducted complementary sensitivity analyses to assess the potential impact of missing outcome data. First, trajectory models were re-estimated using all participants with at least two available outcome measurements (BADL, *n* = 7,769; IADL, *n* = 7,825), thereby allowing incomplete follow-up information to contribute to maximum-likelihood estimation. Second, under a missing-at-random (MAR) assumption, missing follow-up BADL and IADL scores were multiply imputed using chained equations with predictive mean matching. The imputation model included baseline BADL and IADL, the IC deficit score, sociodemographic, lifestyle, and health covariates, as well as observed BADL and IADL measurements from the other follow-up waves. Twenty imputed datasets were generated, and estimates were combined using Rubin’s rules. Third, to examine departures from MAR, we performed pattern-mixture sensitivity analyses under missing-not-at-random (MNAR) assumptions. For observations that were originally missing, the imputed BADL and IADL scores were each shifted downward by 0.5 points in one scenario and by 1.0 point in a second scenario, representing progressively more unfavorable MNAR assumptions. Scores were constrained to remain within the valid scale ranges. The primary IC-by-time interaction estimates were compared across the complete-case, MAR, and MNAR analyses.

#### 2.3.6 Sensitivity analysis using continuous IC measures

To assess whether the longitudinal associations were sensitive to dichotomization of continuous IC measures, we conducted an additional sensitivity analysis retaining baseline MMSE and BMI as continuous variables. MMSE was centered at 24 points. Because the primary vitality definition considered both low and high BMI as deficits, BMI was centered at 21.7 kg/m^2^ and modeled using both linear and quadratic terms to allow for nonlinearity. The remaining four IC domains (locomotion, vision, hearing, and psychological capacity) were retained as binary deficits and summed into a score ranging from 0 to 4. Separate mixed-effects models for BADL and IADL included interactions between time and continuous MMSE, linear and quadratic BMI terms, and the remaining four-domain deficit score, with adjustment for the same covariates as in the primary analysis. This sensitivity analysis included 2,027 participants with complete data for these measures.

## 3 Results

### 3.1 Demographic characteristics

The primary analytical sample comprised 2,045 participants with complete BADL and IADL data at all four waves, representing 14.0% of the 14,658 eligible participants at baseline. Compared with the 12,613 baseline-eligible participants who were not included in the primary complete-case analysis, the 2,045 included participants were younger and had fewer baseline IC deficits, higher BADL and IADL scores, higher educational attainment, a higher proportion who were married/separated, and better self-rated health (all *p* < 0.001; Supplementary Table S19). Ethnicity, physical activity/exercise status, and number of chronic diseases did not differ significantly between the two groups. Of the participants, 1,481 (72.42%) were 60–79 years old, 1,072 (52.42%) were female, 1,912 (93.50%) were Han ethnicity, 1,086 (53.11%) were literacy, 1,262 (61.71%) were married/separated. The mean IC deficit score (Standard deviation, SD) at baseline was 1.50 (1.13). More details are shown in the Supplementary Table S5.

### 3.2 Association between the IC deficit score and physical function trajectories

Using the GBTM, we identified two distinct BADL trajectory groups (Group 1: *High and declining*, 18.1%; Group 2: *High and stable*, 81.9%) (Figure 1). In Group 1, BADL scores were high at baseline and rapidly declined over time, while in Group 2, BADL scores were high at baseline and remained stable over time. The parameters of the GBTM indicated that the model was well-fitted (see Supplementary Table S6). The difference in baseline characteristics between the two trajectory groups is presented in Supplementary Table S5. The results of the generalized linear model showed that older adults with a higher IC deficit score were at a higher risk (adjusted odds ratio, OR: 1.22, 95% CI: 1.09, 1.37, *p* = 0.001) of being assigned to Group 1 after controlling for other confounders (Table 1).

**Figure 1:**
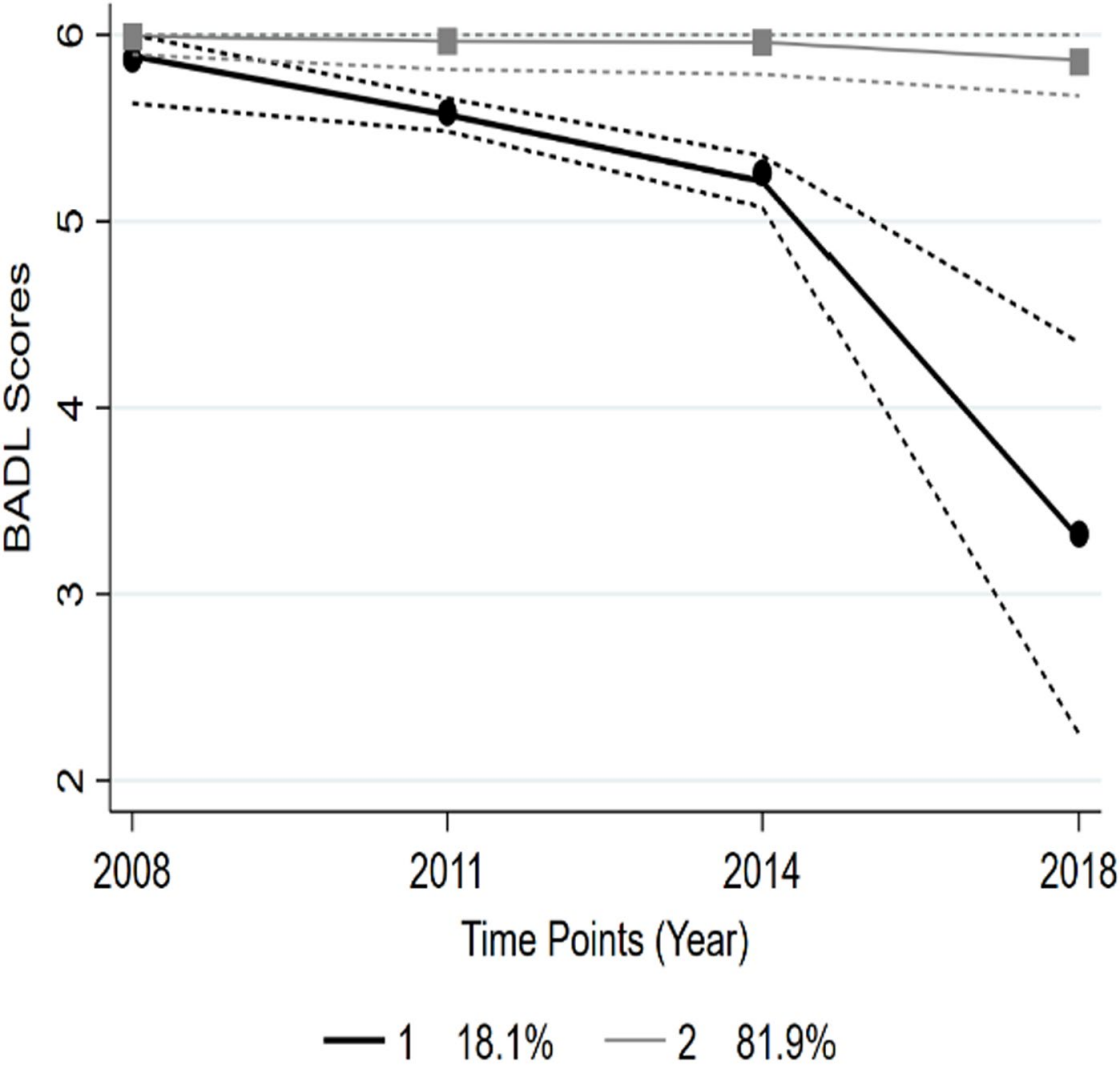
BADL trajectories with 95% confidence intervals estimated in the complete-case survivor cohort (*n* = 2,045); participants with incomplete follow-up BADL or IADL data were excluded. Number of participants contributing observed data at each time point: 2008, *n* = 2,045; 2011, *n* = 2,045; 2014, *n* = 2,045; 2018, *n* = 2,045.

**Table 1:** Association of the IC deficit score with BADL and IADL trajectories.

Characteristics	BADL trajectories^a^		IADL trajectories^b^			
	Group 1 (*High and declining*), OR (95% CI)^c^	*p* value	Group 1 (*Medium and severely declining*), OR (95% CI)^d^	*p* value	Group 2 (*High and moderately declining*), OR (95% CI)^d^	*p* value
IC deficit score	1.22 (1.09, 1.37)	0.001	1.94 (1.66, 2.27)	<0.001	1.37 (1.22, 1.53)	<0.001
Demographic factors						
Age (years old)						
60–79	reference		reference		reference	
≥80	4.62 (3.46, 6.17)	<0.001	23.30 (14.76, 33.69)	<0.001	4.55 (3.43, 6.05)	<0.001
Sex						
Male	reference		reference		reference	
Female	1.77 (1.22, 2.57)	0.003	3.42 (2.08, 5.63)	<0.001	1.72 (1.29, 2.30)	<0.001
Ethnicity						
Han	reference		reference		reference	
Minority	0.92 (0.53, 1.60)	0.777	0.65 (0.32, 1.28)	0.212	0.49 (0.31, 0.77)	0.002
Educational level						
Literacy	reference		reference		reference	
Illiteracy	1.17 (0.86, 1.58)	0.317	2.38 (1.60, 3.52)	<0.001	2.07 (1.65, 2.60)	<0.001
Marital status						
Married/separated	reference		reference		reference	
Divorced/widowed/never married	0.82 (0.61, 1.10)	0.189	1.48 (1.02, 2.17)	0.040	1.51 (1.20, 1.91)	<0.001
Self-rated economic status						
Rich	reference		reference		reference	
Fair	1.06 (0.71, 1.60)	0.763	1.08 (0.63, 1.84)	0.786	1.05 (0.76, 1.46)	0.748
Poor	0.99 (0.59, 1.66)	0.964	0.72 (0.36, 1.42)	0.343	0.99 (0.66, 1.50)	0.963
Lifestyle factors						
Smoking status						
Never	reference		reference		reference	
Ever/present	1.17 (0.81, 1.70)	0.397	1.10 (0.67, 1.82)	0.695	1.21 (0.91, 1.60)	0.192
Alcohol consumption status						
Never	reference		reference		reference	
Ever/present	0.79 (0.57, 1.11)	0.176	0.61 (0.39, 0.96)	0.033	0.94 (0.73, 1.21)	0.626
Physical activity/exercise status						
Yes	reference		reference		reference	
No	1.14 (0.86, 1.51)	0.365	1.01 (0.70, 1.46)	0.941	0.98 (0.79, 1.22)	0.866
Health factors						
Self-rated health						
Good	reference		reference		reference	
Fair	0.87 (0.64, 1.19)	0.389	1.20 (0.81, 1.80)	0.364	1.12 (0.88, 1.41)	0.367
Bad	1.06 (0.70, 1.59)	0.784	3.09 (1.84, 5.19)	<0.001	1.81 (1.27, 2.57)	0.001
Chronic diseases						
0	reference		reference		reference	
1	1.01 (0.73, 1.38)	0.976	1.52 (1.00, 2.30)	0.049	1.10 (0.86, 1.40)	0.458
≥2	1.42 (1.01, 2.01)	0.045	2.30 (1.45, 3.60)	<0.001	1.43 (1.09, 1.88)	0.010

IC deficit score ranges from 0 to 6, with higher scores indicating more impaired domains (worse intrinsic capacity). BADL, Basic Activities of Daily Living; IADL, Instrumental Activities of Daily Living; ^a^Group 2 (High and stable group) as the reference group for the BADL analysis; ^b^Group 3 (High and slightly declining group) as the reference group for the IADL analysis; ^c^Evaluated by a generalized linear model with binomial family and default logit link; ^d^Evaluated by a multinomial logistic regression model.

In terms of IADL, three distinct trajectory groups were identified: Group 1: *Medium and severely declining* (13.0%); Group 2: *High and moderately declining* (41.3%); Group 3: *High and mildly declining* (45.7%) (Figure 2). In Group 1, the IADL scores were medium at baseline but severely declined over time. In Group 2, the IADL scores were high at baseline but immediately declined over time, and in Group 3, the IADL scores were high at baseline and slightly declined over time. The differences in baseline characteristics of the different subgroups of IADL trajectories and fitted parameters of the GBTM are shown in Supplementary Tables S7, S8. Multinomial logistic regression showed that older adults with a higher IC deficit score were at a higher risk of being assigned to the “medium and severely declining” group (OR: 1.94, 95% CI: 1.66, 2.27, *p* < 0.001) or *the “*high and moderately declining” group (OR: 1.37, 95% CI: 1.22, 1.53, *p <* 0.001) after adjustment for other confounders (Table 1).

**Figure 2:**
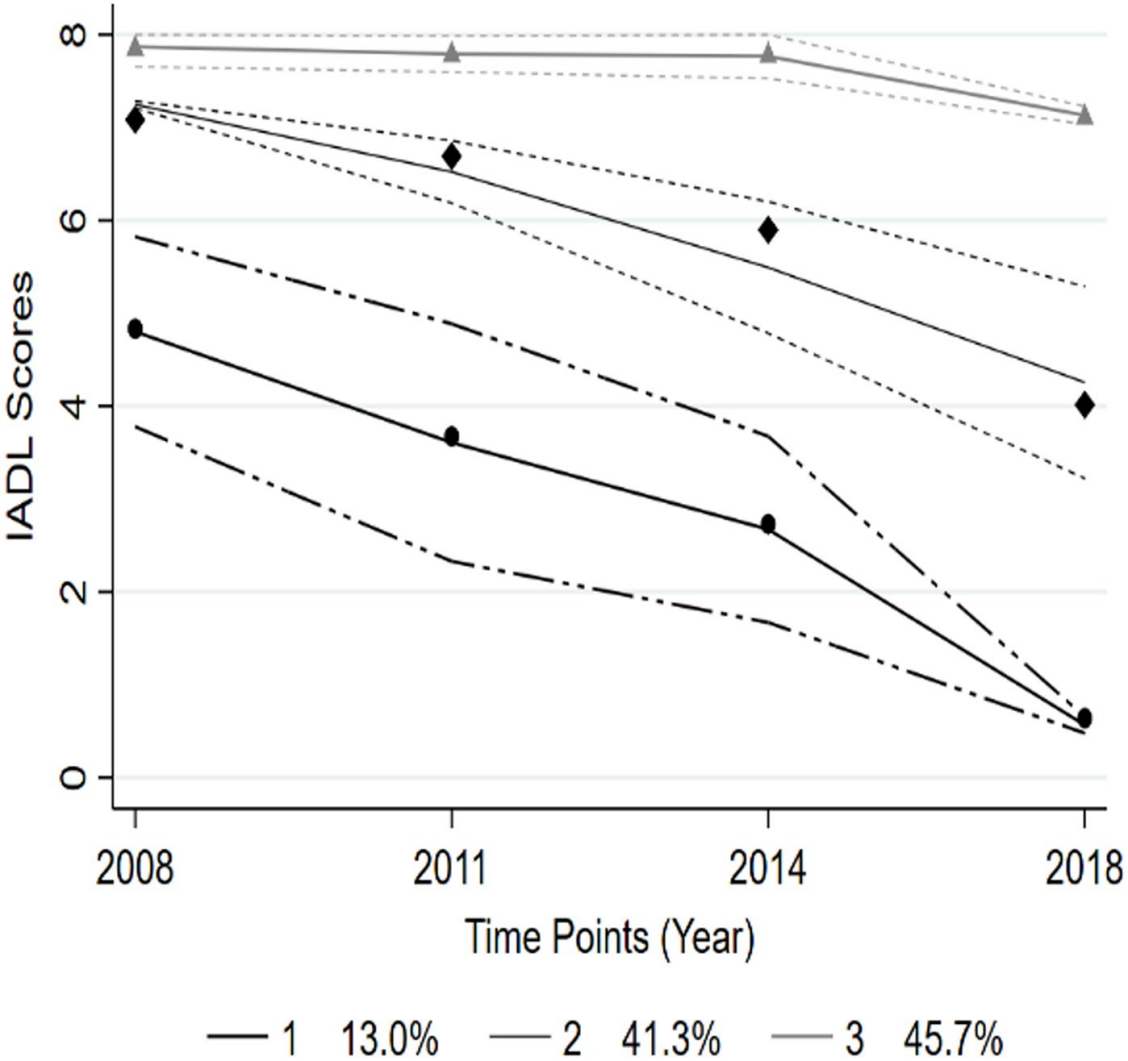
IADL trajectories with 95% confidence intervals estimated in the complete-case survivor cohort (*n* = 2,045); participants with incomplete follow-up BADL or IADL data were excluded. Number of participants contributing observed data at each time point: 2008, *n* = 2,045; 2011, *n* = 2,045; 2014, *n* = 2,045; 2018, *n* = 2,045.

### 3.3 Association between the IC deficit score and annual rates of change in physical function

Table 2 provides an overview of the association between IC and the annual rate of change in physical function. Without consideration of interaction with time, each one-unit increase in the IC deficit score significantly resulted in a decline in BADL and IADL scores by −0.022 (95% CI: −0.033, −0.011, *p* < 0.001) points and −0.332 (95% CI: −0.392, −0.271, *p* < 0.001) points, respectively, after adjustment for all covariates. Similarly, BADL and IADL scores decreased by −0.024 (95% CI: −0.031, −0.017, *p* < 0.001) points and −0.199 (95% CI: −0.221, −0.178, *p* < 0.001) points, respectively, each year after adjustment for all covariates. Regarding the interaction between the IC deficit score and time, each one-unit increase in the baseline IC deficit score was associated with an additional annual decline of 0.007 BADL points (β = −0.007, 95% CI: −0.010 to −0.003, *p* = 0.001). Over the 10-year follow-up, this coefficient corresponds to an additional decline of approximately 0.07 points on the 0–6 BADL scale per one-unit increase in the baseline IC deficit score. The mean annual decline in IADL scores caused by a mean increment in one unit of the IC deficit score was −0.009 (95% CI: −0.021, 0.002, *p* = 0.111) points, but it was not statistically significant.

**Table 2:** Longitudinal associations between the IC deficit score and annual changes in physical function.

Physical function measures^a^	Model terms	Adjusted coefficient (95% CI)^b^	*p* value
BADL	IC	−0.022 (−0.033, −0.011)	<0.001
	Time	−0.024 (−0.031, −0.017)	<0.001
	IC × Time	−0.007 (−0.010, −0.003)	0.001
IADL	IC	−0.332 (−0.392, −0.271)	<0.001
	Time	−0.199 (−0.221, −0.178)	<0.001
	IC × Time	−0.009 (−0.021, 0.002)	0.111

IC deficit score: Intrinsic capacity deficit score (range 0–6, higher = worse capacity); CI: Confidence Interval; BADL: Basic activities of daily living; IADL: Instrumental activities of daily living.

a Higher scores indicate better physical function.

b Adjusted for age, sex, ethnicity, education, marital status, self-rated economic status, smoking status, alcohol consumption status, exercise status, self-rated health, and number of chronic diseases.

### 3.4 Sensitivity analyses

Sensitivity analyses indicated that attrition affected the estimated distribution of physical-function trajectories. When participants with at least two available measurements were included, the proportion assigned to the declining BADL trajectory increased from 18.1% in the complete-case sample to 28.1%, while the proportion assigned to the severe IADL-decline trajectory increased from 13.0 to 25.3%. Nevertheless, the associations between a higher baseline IC deficit score and adverse trajectory membership remained consistent: the adjusted OR for the declining BADL trajectory was 1.34 (95% CI, 1.28–1.40), and the corresponding RRRs were 2.13 (95% CI, 1.99–2.29) for severe and 1.40 (95% CI, 1.32–1.48) for moderate IADL decline.

For the longitudinal mixed-effects analyses, the negative IC-by-time association for BADL remained statistically significant after multiple imputation under MAR and after MNAR delta adjustments of −0.5 and −1.0 points. Under the −1.0-point MNAR scenario, the BADL interaction coefficient was −0.013 (95% CI, −0.020 to −0.007; *p* < 0.001). In contrast, the IADL IC-by-time estimate was sensitive to the handling of missing data: the nonsignificant interaction in the complete-case analysis (β = −0.009, 95% CI, −0.021 to 0.002; *p* = 0.111) became positive after MAR multiple imputation and remained positive in both MNAR scenarios (β = 0.050, 95% CI, 0.041–0.058 under δ = −1.0). Detailed results are presented in Supplementary Table S17.

### 3.5 Results of stratified analysis by age

Additionally, we identified BADL and IADL trajectories by age group. The fitting parameters of GBTM and selected trajectories were demonstrated in Supplementary Tables S9–S14, Supplementary Figures S1, S2. Similar to all included older adults, two distinct BADL trajectories with similar shapes were identified in adults aged 60–79 and ≥80 years old, respectively. Despite the similar numbers and shapes, the proportions belonging to the “high and stable” BADL group were largely different. Adults aged 60–79 had a higher proportion belonging to the “*high and stable*” BADL group than those aged ≥80 (89.6% vs. 44.1%).

Similarly, three distinct IADL trajectories were identified in adults aged 60–79 and ≥80 years old, respectively. The IADL trajectories in adults aged 60–79 were similar to those of all older adults included in the study. However, in those aged ≥80 years, the distinct trajectories were identified with different shapes [Group 1: *Low and declining* (15.6%); Group 2: *Medium and severely declining* (44.2%); Group 3: *Medium and moderately declining* (40.2%)]. In other words, IADL scores were lower at baseline in adults aged ≥80 years and fell more rapidly over time compared to those aged 60–79 years. Multivariable analysis revealed a similar significant association between IC and BADL/IADL trajectories in adults aged 60–79 and ≥80 years, as well as in all included participants, although with slightly different odds ratios (ORs).

Supplementary Tables S15, S16 show the associations between IC and the annual rates of change in BADL and IADL scores stratified by age. Similar to those of all included older adults, without considering the interaction with time, a mean increment in one unit of the IC deficit score significantly resulted in a decline in BADL and IADL scores in both age groups. Such an association was not significant in adults aged 60–79 years but significant in those aged ≥80 years when considering the interaction with time. Additionally, the decline in the annual rate of change in IADL scores was much higher in adults aged ≥80 years than those aged 60–79 years.

### 3.6 Sensitivity analysis using continuous IC measures

In the sensitivity analysis retaining MMSE and BMI as continuous measures (*n* = 2,027), higher baseline MMSE was associated with a slower decline in BADL (MMSE×time: β = 0.0035, 95% CI 0.0023 to 0.0047, *p* < 0.001), whereas the linear and quadratic BMI-by-time terms were not jointly significant (*p* = 0.250). A greater number of deficits in the remaining four IC domains was associated with a faster BADL decline (β = −0.0130, 95% CI − 0.0192 to −0.0069, *p* < 0.001). In contrast, none of these longitudinal interactions was statistically significant for IADL (MMSE×time: β = 0.0014, 95% CI − 0.0014 to 0.0041, *p* = 0.333; joint BMI-by-time test, *p* = 0.288; other four IC deficits×time: β = −0.0085, 95% CI − 0.0223 to 0.0052, *p* = 0.224) (Supplementary Table S18).

## 4 Discussion

Based on 10-year follow-up data from CLHLS, two distinct BADL trajectories and three distinct IADL trajectories were identified among Chinese older adults. In addition, the cohort study found that higher IC deficit score was associated with an accumulated faster decline in BADL but not with a decrease in IADL. Similar results were found in stratified analysis by age group, although with different magnitudes. To our knowledge, this is the first study to explore the value of IC in predicting the longitudinal trajectories of physical function over time and quantify how IC affects longitudinal changes in physical function.

Previous studies have explored the trajectories of physical function in older adults. One study identified three distinct trajectories: a no/little decline group, an intermediate decline group, and a severely declining group, in younger older adults (aged from 60 to 70 years old) (7). Another study identified three subgroups of IADL trajectories: the low-risk, increasing-risk, and high-risk groups (27). Different from their reports, our study identified two distinct groups of BADL trajectories and three distinct trajectories of IADL, respectively, in Chinese older adults. The heterogeneity of included participants and measurement might explain the differences in results. Physical function was evaluated by combining two items of BADL and four items of IADL in older adults aged 60–70 years in the study of Jonkman et al. (7). Using much more comprehensive physical function measurements, our study revealed the heterogeneous developmental trajectories of BADL and IADL over time in older adults. In general, the decline in IADL was much earlier and faster in the early period but slower in the later period than in BADL. Additionally, stratified by age, we found similar results. Importantly, we found that IADL trajectories in older adults, especially in adults aged ≥80 years, declined more severely, indicating more attention should be paid to such a population. The current study’s findings contribute to understanding the natural developing course of physical function and highlight the importance of maintaining and improving optimal physical function in older adults.

Our study expands the existing knowledge on the association between IC and long-term changes in physical function. Previous cross-sectional studies have confirmed the predictive ability of IC for physical function. Campbell et al. (28) found that a 1-unit increase in IC was associated with a 7%–9% reduction in BADL/IADL limitations. Similarly, Stolz et al. (29) reported that a 1-unit decrease in the IC deficit score increased the risk of ADL difficulty by 7% (29). However, physical function is dynamic over time; those studies failed to evaluate the association between IC and longitudinal changes in physical function over time due to the nature of the cross-sectional study. In the primary complete-case mixed-effects analysis, each one-unit increase in the baseline IC deficit score was associated with an additional annual decline of 0.007 points in BADL (β = −0.007, 95% CI − 0.010 to −0.003, *p* = 0.001). In contrast, the IC × time interaction for IADL was not statistically significant (β = −0.009, 95% CI − 0.021 to 0.002, *p* = 0.111). Sensitivity analyses further showed that the IADL interaction estimate was sensitive to assumptions regarding missing follow-up data. The effect of the association between the IC deficit score and the annual rate of change in physical function is small, but a subtle decline can indicate a preclinical decline in physical function. Although the BADL IC × time interaction was statistically significant, the clinical relevance of this finding requires careful interpretation. Our model estimated that each one-unit increment in the IC deficit score was associated with an annual BADL decline of −0.007 points, which translates to approximately 0.07 points over the 10-year follow-up on a 0–6 scale. While no universally accepted minimal clinically important difference (MCID) exists for this specific six-item BADL instrument, studies using comparable functional measures (e.g., the Katz ADL index or the Barthel index) suggest that a change of at least 0.5 to 1.0 points is generally required to reflect a perceptible loss of independence (30). Thus, the 0.07-point decline we observed is an order of magnitude smaller and is unlikely to represent a clinically overt functional loss. Instead, this subtle change is best interpreted as an early, preclinical erosion of functional reserve. From a preventive perspective, detecting such subclinical trajectories is valuable, as it may identify older adults who could benefit from interventions to preserve intrinsic capacity before overt disability develops. It is also worth noting that baseline IC deficit score significantly predicted IADL trajectory membership, whereas the IC × time interaction was not statistically significant for IADL in the mixed-effects model. However, this finding was not robust to alternative assumptions regarding missing follow-up data. This apparent discrepancy likely reflects the different aspects of longitudinal change captured by the two approaches. The trajectory model identifies subgroups with qualitatively distinct long-term decline patterns and tests whether baseline IC predicts assignment to a worse trajectory, reflecting between-person differences in overall functional course. In contrast, the IC × time interaction in the mixed-effects model examines whether baseline IC modifies the linear within-person rate of decline averaged across the sample. For IADL, which involves complex, socially embedded tasks, IC impairment may primarily shift an individual toward a worse trajectory early in the follow-up, but once on that trajectory, the year-to-year slope may be driven more by other factors such as social environment, cognitive decline, or acute health events. Furthermore, the analytic sample comprised older adults who survived and remained in the study for 10 years, likely representing a group with relatively preserved IADL function; this restricted variability in within-person IADL slopes may have reduced the statistical power to detect a significant IC × time interaction. Importantly, this finding does not undermine the overall conclusion that higher IC deficit scores are associated with worse physical function outcomes; rather, it suggests that IC primarily differentiates long-term functional trajectories rather than accelerating the annual rate of IADL decline in this cohort. An additional sensitivity analysis retaining MMSE and BMI as continuous measures provided partial support for the BADL findings. Higher baseline MMSE was associated with a slower annual decline in BADL, whereas the linear and quadratic BMI-by-time terms were not jointly significant. In contrast, none of the corresponding time-interaction terms for MMSE, BMI, or the remaining four-domain deficit score was statistically significant for IADL, indicating that the IADL findings were more sensitive to the operationalization of the IC domains (Supplementary Table S18). Additionally, the results of stratified analysis by age showed that the decrease in the annual rate of change in IADL scores was much higher in adults aged ≥80 years than those aged 60–79 years. From this aspect, health professionals and policymakers should pay attention to IC in older adults, particularly those aged ≥80 years. Previous studies have pointed out that impairment of a single component of IC, such as cognitive impairment, depressive symptoms, and visual or hearing loss, could limit physical function (31–33). However, the five overarching IC domains—cognition, locomotion, sensory capacity, vitality, and psychological capacity—are considered interrelated and inseparable (33). In the present study, sensory capacity was operationalized as two separate domains, visual and hearing capacities, resulting in six measured IC domains. The interaction of six domains contributes to the accumulated changes in physical function over time.

Our study provides additional evidence on the predictive value of IC in long-term changes in physical function. It highlights the importance of IC in monitoring and preventing physical function decline. On one hand, we suggest a standardized measurement to assess each domain of IC should be developed. On the other hand, we strongly recommend that routine and comprehensive assessment of IC and early detection of people with lower IC are critical, especially for adults aged ≥80 years, because they might provide an opportunity for primary and secondary prevention in older adults. Moreover, our findings might help develop effective multidimensional interventions such as boosting multicomponent exercise programs, nutrition, sleep management, pharmacological treatments, etc. (35, 36) to improve IC for maintaining physical function and achieving healthy aging in older adults.

### 4.1 Strengths and limitations

This study’s main strength was using a representative national cohort study to identify the trajectories of physical function and to examine the association between IC and physical function trajectories. Additionally, we conducted a stratified analysis by age, which provides deeper insights into physical function trajectories and their association with IC. However, some limitations of the study need to be acknowledged. First, the primary trajectory analyses were restricted to 2,045 participants with complete observations across all four waves, representing approximately 14% of the eligible baseline cohort. Participants retained throughout follow-up differed substantially from those not included in the primary complete-case analysis, with the retained participants generally showing a more favorable baseline functional profile. This selection was also evident in a direct baseline comparison: compared with baseline-eligible participants who were not included in the primary complete-case analysis, included participants had fewer IC deficits and substantially better baseline BADL and IADL function, in addition to being younger and having more favorable educational, marital, and self-rated health profiles (Supplementary Table S19). Our sensitivity analyses support the expected direction of this selection: when participants with incomplete follow-up but at least two available measurements were included, the estimated proportions of the declining BADL trajectory and severe IADL-decline trajectory increased from 18.1% to 28.1% and from 13.0% to 25.3%, respectively. Thus, the complete-case analysis likely underestimated the absolute burden of functional decline in the baseline population. Reassuringly, the associations between a higher baseline IC deficit score and adverse BADL and IADL trajectory membership remained similar or slightly stronger in the all-available analyses, and the BADL IC-by-time association remained robust under both MAR multiple imputation and prespecified MNAR delta-adjustment scenarios. However, the IC-by-time estimate for IADL changed in direction and statistical significance after imputation, indicating that inference regarding the linear annual IADL slope is sensitive to the assumptions used to handle missing follow-up data. Therefore, residual selection bias due to informative dropout and mortality cannot be excluded, and the absolute trajectory distributions and longitudinal IADL slope should be interpreted cautiously. Second, although we constructed the IC deficit score following the WHO ICOPE framework, the specific measures were adapted from items available in the CLHLS questionnaire rather than derived from a standardized and validated IC assessment instrument (37). For instance, psychological capacity was assessed by only two self-reported items on anxiety and loneliness rather than a validated multi-item scale; visual capacity depended on the participant’s ability to identify a break in a circle shown by an interviewer; and hearing capacity relied on the interviewer’s judgment of whether the participant could hear the conversation. This use of simplified proxy measures may limit construct validity. These simplified proxy measures are susceptible to reporting and exposure misclassification bias. Psychological capacity was based on two self-reported items, visual capacity depended on participant responses during an interviewer-administered task, and hearing capacity relied partly on interviewer judgment. If this misclassification was predominantly non-differential with respect to subsequent physical-function outcomes, it would be expected to attenuate the observed associations toward the null. However, the direction and magnitude of the bias cannot be determined with certainty if misclassification differed according to participants’ health or functional status. Nevertheless, our operationalization is consistent with several prior longitudinal studies that used CLHLS data to examine IC and health outcomes (24), which supports its face validity for capturing the overall IC construct. Future studies should employ more comprehensive, validated instruments specifically designed for IC assessment and incorporate more objective measures for the psychological, visual, and hearing domains, such as validated depression and anxiety scales, audiometry, and standardized visual acuity tests. Third, although BADL and IADL are widely used indicators of functional status in epidemiological studies, they primarily assess the independence dimension of physical function and do not directly capture other important aspects such as muscle strength, balance, or gait speed. In particular, the CLHLS IADL measure combines traditional instrumental tasks (e.g., shopping, cooking, using public transportation) with physical performance tasks (e.g., walking 1 km, lifting 5 kg, crouching and standing three times). While this hybrid composition may enhance sensitivity to early functional decline, it should be considered when comparing results with studies using purely instrumental scales such as the Lawton-Brody IADL instrument. Additionally, some conceptual overlap exists between the IC domains and the outcome measures. Specifically, the locomotor capacity domain of IC (assessed by picking up a book from the floor, standing up from a chair, and turning 360°) shares conceptual ground with the physical performance tasks in the CLHLS IADL measure. This overlap may partially contribute to the observed association between the IC deficit score and IADL decline. However, it should be noted that a significant association was also observed between the IC deficit score and BADL trajectories, and BADL comprises only self-care activities (e.g., bathing, dressing, eating) with no physical performance tasks. This suggests that the relationship between IC and functional decline is not solely attributable to measurement overlap. Therefore, the selected outcome measures may not comprehensively reflect the full spectrum of physical function. Future studies could incorporate additional performance-based measures to provide a more multidimensional assessment. Fourth, because this is an observational study, residual and unmeasured confounding cannot be ruled out. Although we adjusted for a broad set of sociodemographic, lifestyle, and health characteristics, information on potentially relevant factors—such as medication use, household income, and availability of care or social support—was not available in the CLHLS dataset. Each of these factors could plausibly influence both IC and functional decline (24). Consequently, the reported associations should be viewed as robust statistical relationships rather than direct causal effects. Therefore, a more objective measurement, including biological [i.e., inflammatory biomarkers (34, 37, 38)], clinical and metabolic biomarkers [i.e., hormonal and cardiorespiratory fitness (39)], and digital [i.e., hearing or eye examination (39)] domains for IC, is needed in the future. Therefore, the reported findings should be interpreted as observational associations rather than causal effects.

## 5 Conclusion

In conclusion, we identified two distinct trajectories of BADL and three distinct trajectories of IADL in older adults. A higher baseline IC deficit score was associated with membership in adverse BADL and IADL trajectory groups and with a small acceleration in annual BADL decline. The estimated annual effect was statistically significant but too small to represent an overt loss of independence at the individual level; it may instead reflect preclinical functional vulnerability. These findings highlight the potential value of regular IC assessment as an early indicator for healthy aging, and support the importance of maintaining intrinsic capacity in Chinese older adults. Effective interventions are needed to manage IC to improve physical function and healthy aging.

## Data Availability

The datasets analyzed for this study are publicly available in the Chinese Longitudinal Healthy Longevity Survey (CLHLS) repository at https://opendata.pku.edu.cn/dataset.xhtml?persistentId=doi:10.18170/DVN/WBO7LK. The original contributions presented in the study are included in the article/Supplementary material; further inquiries can be directed to the corresponding authors.
