# Supplementary material for: Association between intrinsic capacity and trajectories of physical function among older adults: findings from a 10-year longitudinal cohort study
Source: Front Public Health. 2026 Sep 23;14:1893548. doi: 10.3389/fpubh.2026.1893548 (PMC13642007; doi:10.3389/fpubh.2026.1893548)
Supplement: Supplementary file 1 [file Supplementary_File_1.docx]

**Contents of the supplemental**

[e-Flowchart of participant selection and sensitivity-analysis samples in the CLHLS, 2008–2018	3](#_Toc186384555)

[Table S1 Comparison of included and excluded participants at each follow-up wave	4](#_Toc186384572)

[Table S2 Descriptive statistics of physical function at different waves in the CLHLS cohort	6](#_Toc186384556)

[Table S3 Measures and codes of intrinsic capacity in the study	7](#_Toc186384557)

[Table S4 Codes of covariates in the study	8](#_Toc186384558)

[Table S5 Distribution differences by trajectory groups of BADL	9](#_Toc186384559)

[Table S6 Fitting parameters for BADL GBTM according to the number of groups and trajectory shapes in older adults	11](#_Toc186384560)

[Table S7 Distribution differences by trajectory groups of IADL	12](#_Toc186384561)

[Table S8 Fitting parameters for IADL GBTM according to the number of groups and trajectory shapes in older adults	14](#_Toc186384562)

[Table S9 Fitting parameters for BADL GBTM according to the number of groups and trajectory shapes in adults aged 60-79 years old	16](#_Toc186384563)

[Table S10 Fitting parameters for BADL GBTM according to the number of groups and trajectory shapes in adults aged 80+ years old	18](#_Toc186384564)

[Fig S1 BADL trajectories in older adults by age groups	17](#_Toc186384565)

[Table S11 Fitting parameters for IADL GBTM according to the number of groups and trajectory shapes in adults aged 60-79 years old	19](#_Toc186384566)

[Table S12 Fitting parameters for IADL GBTM according to the number of groups and trajectory shapes in adults aged 80+ years old	2](#_Toc186384567)0

[Fig S2 IADL trajectories in older adults by age groups	21](#_Toc186384568)

[Table S13 Association of IC with trajectories of BADL and IADL in adults aged 60-79 years old	22](#_Toc186384569)

[Table S14 Association of IC with trajectories of BADL and IADL in adults aged 80+ years old	24](#_Toc186384570)

[Table S15 Longitudinal associations between IC and the annual rate of change in physical function in adults aged 60-79 years old	26](#_Toc186384571)

[Table S16 Longitudinal associations between IC and the annual rate of change in physical function in adults aged 80+ years old	27](#_Toc186384572)

[Table S17 Sensitivity analyses addressing attrition and missing follow-up data	28](#_Toc186384572)

[Table S18 Sensitivity analysis retaining MMSE and BMI as continuous measures	3](#_Toc186384572)0

Table S19 Baseline characteristics of participants included and not included in the primary complete-case analysis.............................................31

# e-Flowchart of participant selection and sensitivity-analysis samples in the CLHLS, 2008–2018

Sensitivity-analysis cohorts

BADL, n=7,769; IADL, n=7,825

MAR/MNAR multiple-imputation analysis:
n=14,658; 20 imputations

Included old adults in the CLHLS 2008 (n=14,658)

((

Excluded because of missing value in BADL/IADL in follow-up (n=7,046)

Screened for eligibility in 2011

(n=7,612)

Excluded because of missing value in BADL/IADL in follow-up (n=3,020)

Screened for eligibility in 2014

(n=4,592)

Excluded because of missing value in BADL/IADL in follow-up (n=2,547)

Finally included for analyzing (BADL/IADL trajectories, and logistic regression)

(n=2,045)

| Table S1 comparison of included and excluded participants in each follow-up wave | | | | | | | | | | | | | | | | | | | | | | | | | | | | | | | | |  |  |
| --- | --- | --- | --- | --- | --- | --- | --- | --- | --- | --- | --- | --- | --- | --- | --- | --- | --- | --- | --- | --- | --- | --- | --- | --- | --- | --- | --- | --- | --- | --- | --- | --- | --- | --- |
| **Baseline Characteristics** | | **2011** | | | | | | | | **2014** | | | | | | | | | | **2018** | | | | | | | | | | | | |  |  |
|  |  | **Excluded  (n=7,046)** | | | **Included (n=7,612)** | | ***P*** | | | **Excluded  (n=3,020)** | | | **Included (n=4,592)** | | | ***P*** | | | | **Excluded  (n=2,547)** | | | | **Included (n=2,045)** | | | | | ***P*** | | | |  |  |
| **Age (years old), N (%)** | |  | | |  | |  | | |  | | |  | | |  | | | |  | | | |  | | | | |  | | | |  |  |
|  | 60-79 | | | 1,202 (17.06) | 3,286 (43.17) | | | <0.001 | | | 749 (24.80) | | 2,537 (55.25) | | | | <0.001 | | | | 1,056 (41.46) | | | | 1,481 (72.42) | | | | | <0.001 | | | |  |
|  | 80+ | | | 5,844 (82.94) | 4,326 (56.83) | | |  |  |  | 2,271 (75.20) | | 2,055 (44.75) | | | |  |  |  |  | 1,491 (58.54) | | | | 564 (27.58) | | | | |  |  |  |  |  |
| **Gender, N (%)** | |  | | |  | |  | | |  | | |  | | |  | | | |  | | | |  | | | | |  | | | |  |  |
|  | Male | | | 3,028 (42.97) | 3,545 (46.57) | | | <0.001 | | | 1,379 (45.66) | | 2,166 (47.17) | | | | 0.197 | | | | 1,193 (46.84) | | | | 973 (47.58) | | | | | 0.618 | | | |  |
|  | Female | | | 4,018 (57.03) | 4,067 (53.43) | | |  |  |  | 1,641 (54.34) | | 2,426 (52.83) | | | |  |  |  |  | 1,354 (53.16) | | | | 1,072 (52.42) | | | | |  |  |  |  |  |
| **Ethnicity, N (%)** | |  | | |  | |  | | |  | | |  | | |  | | | |  | | | |  | | | | |  | | | |  |  |
|  | Han | | | 6,584 (93.44) | 7,141 (93.81) | | | 0.360 | | | 2,844 (94.17) | | 4,297 (93.58) | | | | 0.291 | | | | 2,385 (93.64) | | | | 1,912 (93.50) | | | | | 0.844 | | | |  |
|  | Minority | | | 462 (6.56) | 471 (6.19) | | |  |  |  | 176 (5.83) | | 295 (6.42) | | | |  |  |  |  | 162 (6.36) | | | | 133 (6.50) | | | | |  |  |  |  |  |
| **Education level, N (%)** | |  | | |  | |  | | |  | | |  | | |  | | | |  | | | |  | | | | |  | | | |  |  |
|  | Literacy | | | 2,484 (35.25) | 3,413 (44.84) | | | <0.001 | | | 1,196 (39.60) | | 2,217 (48.28) | | | | <0.001 | | | | 1,131 (44.41) | | | | 1,086 (53.11) | | | | | <0.001 | | | |  |
|  | Illiteracy | | | 4,562 (64.75) | 4,199 (55.16) | | |  |  |  | 1,824 (60.40) | | 2,375 (51.72) | | | |  |  |  |  | 1,416 (55.59) | | | | 959 (46.89) | | | | |  |  |  |  |  |
| **Marital status, N (%)** | |  | | |  | |  | | |  | | |  | | |  | | | |  | | | |  | | | | |  | | | |  |  |
|  | Married/separated | | | 1,738 (24.67) | 3,456 (45.40) | | | <0.001 | | | 1,033 (34.21) | | 2,423 (52.77) | | | | <0.001 | | | | 1,161 (45.58) | | | | 1,262 (61.71) | | | | | <0.001 | | | |  |
|  | Divorced/widowed/never married | | | 5,308 (75.33) | 4156 (54.60) | | |  |  |  | 1,987 (65.79) | | 2,169 (47.23) | | | |  |  |  |  | 1,386 (54.42) | | | | 783 (38.29) | | | | |  |  |  |  |  |
| **Self-rated economic status, N (%)** | |  | | |  | |  | | |  | | |  | | |  | | | |  | | | |  | | | | |  | | | |  |  |
|  | Rich | | 941 (13.36) | | | 1,058 (13.90) | | | 0.442 | | | 395 (13.08) | | 663 (14.44) | | | | 0.018 | | | | | 399 (15.67) | | | | 264 (12.91) | | | | 0.006 | | | |
|  | Fair | | 4,861 (68.99) | | | 5,258 (69.08) | | |  |  |  | 2,070 (68.54) | | 3,188 (69.43) | | | |  |  |  |  |  | 1,721 (67.57) | | | | 1,467 (71.74) | | | |  |  |  |  |
|  | Poor | | 1,244 (17.66) | | | 1,296 (17.03) | | |  |  |  | 555 (18.38) | | 741 (16.14) | | | |  |  |  |  |  | 427 (16.76) | | | | 314 (15.35) | | | |  |  |  |  |
| **Smoking, N (%)** | |  | | |  | |  | | |  | | |  | | |  | | | |  | | | |  | | | | |  | | | |  |  |
|  | No | | 4,789 (67.97) | | | 4,854 (63.77) | | | <0.001 | | | 1,958 (64.83) | | | 2,896 (63.07) | | | | 0.116 | | | | 1,604 (62.98) | | | | | 1,292 (63.18) | | | | 0.888 | | |
|  | Yes | | 2,257 (32.03) | | | 2,758 (36.23) | | |  |  |  | 1,062 (35.17) | | | 1,696 (36.93) | | | |  |  |  |  | 943 (37.02) | | | | | 753 (36.82) | | | |  |  |  |
| **Alcohol assumption, N (%)** | |  | | |  | |  | | |  | | |  | | |  | | | |  | | | |  | | | | |  | | | |  |  |
|  | No | | 4,939 (70.10) | | | 5,094 (66.92) | | | <0.001 | | | 2,072 (68.61) | | | 3,022 (65.81) | | | | 0.011 | | | | 1,711 (67.18) | | | | | 1,311 (64.11) | | | | 0.029 | | |
|  | Yes | | 2,107 (29.90) | | | 2,518 (33.08) | | |  |  |  | 948 (31.39) | | | 1,570 (34.19) | | | |  |  |  |  | 836 (32.82) | | | | | 734 (35.89) | | | |  |  |  |
| **Physical activity/exercise, N (%)** | |  | | |  | |  | | |  | | |  | | |  | | | |  | | | |  | | | | |  | | | |  |  |
|  | No | | 4,244 (60.23) | | | 4,257 (55.92) | | | <0.001 | | | 1,711 (56.66) | | 2,546 (55.44) | | | | | 0.298 | | | | 1,387 (54.46) | | | | 1,159 (56.67) | | | | 0.133 | | | |
|  | Yes | | 2,802 (39.77) | | | 3,355 (44.08) | | |  |  |  | 1,309 (43.34) | | 2,046 (44.56) | | | | |  |  |  |  | 1,160 (45.54) | | | | 886 (43.33) | | | |  |  |  |  |
| **Self-rated health, N (%)** | |  | | |  | |  | | |  | | |  | | |  | | | |  | | | |  | | | | |  | | | |  |  |
|  | Good | | 3,203 (45.46) | | | 4,017 (52.77) | | | <0.001 | | | 1,500 (49.67) | | | 2,517 (54.81) | | | | <0.001 | | | | 1,354 (53.16) | | | | 1,163 (56.87) | | | | 0.039 | | | |
|  | Fair | | 2,550 (36.19) | | | 2,503 (32.88) | | |  |  |  | 1,045 (34.60) | | | 1,458 (31.75) | | | |  |  |  |  | 843 (33.10) | | | | 615 (30.07) | | | |  |  |  |  |
|  | Bad | | 1,293 (18.35) | | | 1,092 (14.35) | | |  |  |  | 475 (15.73) | | | 617 (13.44) | | | |  |  |  |  | 350 (13.74) | | | | 267 (13.06) | | | |  |  |  |  |
| **Chronic diseases, N (%)** | |  | | |  | |  | | |  | | |  | | |  | | | |  | | | |  | | | | |  | | | |  |  |
|  | 0 | | 3,047 (43.24) | | | 3,256 (42.77) | | | 0.588 | | | 1,301 (43.08) | | | 1,955 (42.57) | | | | 0.830 | | | 1,067 (41.89) | | | | 888 (43.42) | | | | | 0.416 | | | |
|  | 1 | | 2,177 (30.90) | | | 2,412 (31.69) | | |  |  |  | 945 (31.29) | | | 1,467 (31.95) | | | |  |  |  | 813 (31.92) | | | | 654 (31.98) | | | | |  |  |  |  |
|  | ≥2 | | 1,822 (25.86) | | | 1,944 (25.54) | | |  |  |  | 774 (25.63) | | | 1,170 (25.48) | | | |  |  |  | 667 (26.19) | | | | 503 (24.60) | | | | |  |  |  |  |

| Table S2 Descriptive statistics of physical function at different waves in the CLHLS cohort | | |
| --- | --- | --- |
| **Waves** | **BADL  Mean (SE)** | **IADL  Mean (SE)** |
| 2008 (wave1, year 2008) | 5.97 (0.01) | 7.14 (0.04) |
| 2011 (wave2, year 2011) | 5.90 (0.01) | 6.80 (0.04) |
| 2014 (wave3, year 2014) | 5.83 (0.02) | 6.34 (0.05) |
| 2018 (wave4, year 2018) | 5.40 (0.03) | 4.99 (0.07) |

# BADL: Basic Activities of Daily Living; IADL: Instrumental Activities of Daily Living; SE: Standard Error.

| Table S3 Measures and codes of intrinsic capacity in the study | | |
| --- | --- | --- |
| **Variables** | **Measures** | **Codes** |
| Cognitive capacity | The Chinese version of MMSE | 1=MMSE<24  0=MMSE≥24 |
| Psychological capacity | (1) Do you often feel fearful or anxious? [1= “Always” OR “Often” OR “Sometimes” OR “Seldom” (Impaired); 0= “Never” (Not impaired)]  (2) Do you often feel lonely and isolated? [1= “Always” OR “Often” OR “Sometimes” OR “Seldom” (Impaired); 0= “Never” (Not impaired)] | 1=Any impaired  0=Without any impaired |
| locomotor capacity | (1) Was the interviewee able to pick up a book from the floor? [1=No (“Impaired”); 0=Yes (Not impaired”)]  (2) Can the interviewee stand up from sitting in a chair? [1=No (“Impaired”); 0=Yes (Not impaired”)]  (3) Was the interviewee able to turn around 360° without help? [1=No (“Impaired”); 0=Yes (Not impaired”)] | 1=Any impaired  0=Without any impaired |
| Vitality | BMI | 1=BMI<18.5 OR BMI>24.9  0=BMI [18.5, 24.9] |
| Visual capacity | Can the interviewee see a break in the circle on the cardboard sheet when lit by a flashlight and distinguish where the break is located? | 1= “Can see only but can’t distinguish” OR “Can’t see” OR “Blind”  0= “Can see and distinguish” |
| Hearing capacity | Was the interviewee able to hear what you said? | 1= “Yes, but needs hearing aid” OR “Partly, despite hearing aid” OR “No”  0= “Yes, without a hearing aid” |

| Table S4 Codes of covariates in the study | |
| --- | --- |
| **Variables** | **Measures** |
| Age | 0=60-79 years old; 1=80+ years old |
| Gender | 0=Male; 1=Female |
| Ethnicity | 0=Han; 1=Minority |
| Educational level | 0=Literacy; 1=Illiteracy |
| Marital status | 0=Married/separated; 1=Divorced/widowed/never married |
| Self-rated economic status | 0=Rich; 1=Fair; 2=Poor |
| Smoking | 0=Never; 1=Ever/present |
| Alcohol assumption | 0=Never; 1=Ever/present |
| Physical activity/exercise | 0=Ever/present yes; 1=Ever and past no |
| Self-rated health | 0=Very good/good; 1=Fair; 2=Bad/very bad |
| Chronic diseases | 0=0; 1=1; 2=2+ |

|  | Table S5 Distribution differences by trajectory groups of BADL | | | | | | | | | |
| --- | --- | --- | --- | --- | --- | --- | --- | --- | --- | --- |
|  |  | | **Total** | | **Group 1  (High, decline)** | | **Group 2  (High, stable)** | | ***P*** | |
|  | **Sample size, N (%)** | | 2,045 (100) | | 294 (14.38) | | 1,751 (85.62) | |  | |
|  | **IC deficit score, Mean (SD)** | | 1.50 (1.13) | | 1.72 (0.06) | | 1.29 (0.02) | | <0.001 | |
|  | **Age (years old), N (%)** | |  | |  | |  | |  | |
| 60-79 | | 1,481 (72.42) | | 120 (40.82) | | 1,361 (77.73) | | <0.001 | |  |
| ≥80 | | 564 (27.58) | | 174 (59.18) | | 390 (22.27) | |  |  |  |
|  | **Gender, N (%)** | |  | |  | |  | |  | |
| Male | | 973 (47.58) | | 94 (31.97) | | 879 (50.20) | | <0.001 | |  |
| Female | | 1072 (52.42) | | 200 (68.03) | | 872 (49.80) | |  |  |  |
|  | **Ethnicity, N (%)** | |  | |  | |  | |  | |
| Han | | 1,912 (93.50) | | 276 (93.88) | | 1,636 (93.43) | | 0.775 | |  |
| Minority | | 133 (6.50) | | 18 (6.12) | | 115 (6.57) | |  |  |  |
|  | **Education level, N (%)** | |  | |  | |  | |  | |
| Literacy | | 1,086 (53.11) | | 113 (38.44) | | 973 (55.57) | | <0.001 | |  |
| Illiteracy | | 959 (46.89) | | 181 (61.56) | | 778 (44.43) | |  |  |  |
|  | **Marital status, N (%)** | |  | |  | |  | |  | |
| Married/separated | | 1,262 (61.71) | | 145 (49.32) | | 1,117 (63.79) | | <0.001 | |  |
| Divorced/widowed/never married | | 783 (38.29) | | 149 (50.68) | | 634 (36.21) | |  |  |  |
|  | **Self-rated economic status, N (%)** | |  | |  | |  | |  | |
| Rich | | 264 (12.91) | | 37 (12.59) | | 227 (12.96) | | 0.878 | |  |
| Fair | | 1,467 (71.74) | | 209 (71.09) | | 1,258 (71.84) | |  |  |  |
| Poor | | 314 (15.35) | | 48 (16.33) | | 266 (15.19) | |  |  |  |
|  | **Smoking, N (%)** | |  | |  | |  | |  | |
| Never | | 1,292 (63.18) | | 212 (72.11) | | 1,080 (61.68) | | 0.001 | |  |
| Ever/present | | 753 (36.82) | | 82 (27.89) | | 671 (38.32) | |  |  |  |
|  | **Alcohol assumption, N (%)** | |  | |  | |  | |  | |
| Never | | 1,311 (64.11) | | 216 (73.47) | | 1,095 (62.54) | | <0.001 | |  |
| Ever/present | | 734 (35.89) | | 78 (26.53) | | 656 (37.46) | |  |  |  |
|  | **Physical activity/exercise, N (%)** | |  | |  | |  | |  | |
| Never | | 1159 (56.67) | | 168 (57.14) | | 991 (56.60) | | 0.861 | |  |
| Ever/present | | 886 (43.43) | | 126 (42.86) | | 760 (43.40) | |  |  |  |
|  | **Self-rated health, N (%)** | |  | |  | |  | |  | |
| Good | | 1,163 (56.87) | | 163 (55.44) | | 1,000 (57.11) | | 0.190 | |  |
| Fair | | 615 (30.07) | | 83 (28.23) | | 532 (30.38) | |  |  |  |
| Bad | | 267 (13.06) | | 48 (16.33) | | 219 (12.51) | |  |  |  |
|  | **Chronic diseases, N (%)** | |  | |  | |  | |  | |
|  | 0 | | 888 (43.32) | | 118 (40.14) | | 770 (43.97) | | 0.130 | |
|  | 1 | | 654 (31.98) | | 90 (30.61) | | 564 (32.21) | |  |  |
|  | ≥2 | | 503 (24.60) | | 86 (29.25) | | 417 (23.81) | |  |  |
|  | BADL: Basic Activities of Daily Living; SD: Standard deviation. | | | | | | | | | |

| Table S6 Fitting parameters for BADL GBTM according to the number of groups and trajectory shapes in older adults | | | | | | | |  |  |
| --- | --- | --- | --- | --- | --- | --- | --- | --- | --- |
| **Number of groups** | **Trajectory shapes^1^** | **BIC^2^** | **BIC^3^** | **AvePP** | | | **OCC** | | |
|  |  |  |  | **Group 1** | **Group 2** | **Group 3** | **Group 1** | **Group 2** | **Group 3** |
| 2 | 3 3 | -3683.16 | -3676.22 | 0.876 | 0.935 | \ | 312.212 | 130.509 | \ |
| 2 | 3 0 | -3687.10 | -3682.24 | 0.895 | 0.905 | \ | 211.723 | 106.021 | \ |
| 2 | 2 3 | -3683.63 | -3677.39 | 0.866 | 0.934 | \ | 287.804 | 129.062 | \ |
| 2 | 2 1 | -3681.22 | -3676.37 | 0.881 | 0.93 | \ | 319.916 | 121.437 | \ |
| 2 | 2 0 | -3691.32 | -3687.16 | 0.916 | 0.896 | \ | 274.147 | 94.997 | \ |
| 2 | 1 1 | -3683.80 | -3679.64 | 0.859 | 0.928 | \ | 264.003 | 117.523 | \ |
| 2 | 1 0 | -3693.10 | -3689.64 | 0.903 | 0.894 | \ | 223.336 | 95.423 | \ |
| 2 | 0 1 | -3775.59 | -3772.12 | 0.784 | 0.975 | \ | 558.948 | 300.548 | \ |
| 2 | 0 0 | -4000.92 | -3998.15 | 0.794 | 0.862 | \ | 124.445 | 62.255 | \ |
| 3 | 3 1 0 | 3685.08 | -3678.15 | 0.763 | 0.658 | 0.732 | 178.400 | 52.237 | 37.349 |
| 3 | 1 3 2 | -3686.23 | -3677.91 | 0.766 | 0.798 | 0.81 | 107.699 | 250.250 | 51.618 |
| 3 | 1 1 2 | -3681.83 | -3674.9 | 0.883 | 0.783 | 0.854 | 486.234 | 137.893 | 66.838 |
| 3 | 1 1 1 | -3679.22 | -3672.98 | 0.641 | 0.878 | 0.942 | 240.187 | 491.197 | 145.973 |
| 3 | 1 1 0 | -3682.05 | -3676.51 | 0.681 | 0.866 | 0.874 | 96.846 | 395.981 | 75.495 |
| 3 | 0 2 2^b^ | -3699.35 | -3692.41 | 0.692 | 0.948 | 0.883 | 1999.084 | 439.834 | 86.266 |
| ^1^Trajectory shapes; 0=zero-order; 1=linear; 2=quadratic; 3=cubic; ^2^BIC=Bayesian information criterion (for the total number of participants); ^3^BIC=Bayesian information criterion (for the total number of observations); AvePP: Average posterior probabilities; OCC: Odds of correct classification; ^a^The final model was chosen for trajectory analysis after a comprehensive comparison of BIC, AvePP, and OCC; ^b^One or more of the groups had a very small proportion of the observations (less than 5%). | | | | | | | | | |

|  | Table S7 Distribution differences by trajectory groups of IADL | | | | | | | | | | | |
| --- | --- | --- | --- | --- | --- | --- | --- | --- | --- | --- | --- | --- |
|  |  | | **Total** | | **Group 1**  **(Medium, severe decline)** | | **Group 2  (High, intermediate decline)** | | **Group 3  (High, slight decline)** | | ***P*** | |
|  | **Sample size, N (%)** | | 2045 (100) | | 250 (12.22) | | 847 (41.42) | | 948 (46.36) | |  | |
|  | **IC deficit score, Mean (SD)** | | 1.50 (0.02) | | 2.43 (1.41) | | 1.63 (1.11) | | 1.13 (0.87) | | <0.001; 1>2>3 | |
|  | **Age (years old), N (%)** | |  | |  | |  | |  | |  | |
| 60-79 | | 1,481 (72.42) | | 69 (27.60) | | 552 (65.17) | | 860 (90.72) | | <0.001 | |  |
| ≥80 | | 564 (27.58) | | 181 (72.40) | | 295 (34.83) | | 88 (9.28) | |  |  |  |
|  | **Gender, N (%)** | |  | |  | |  | |  | |  | |
| Male | | 973 (47.58) | | 50 (20.00) | | 339 (40.02) | | 584 (61.60) | | <0.001 | |  |
| Female | | 1072 (52.42) | | 200 (80.00) | | 508 (59.98) | | 364 (38.40) | |  |  |  |
|  | **Ethnicity, N (%)** | |  | |  | |  | |  | |  | |
| Han | | 1,912 (93.50) | | 232 (92.80) | | 806 (95.16) | | 874 (92.19) | | 0.035 | |  |
| Minority | | 133 (6.50) | | 18 (7.20) | | 41 (4.84) | | 74 (7.81) | |  |  |  |
|  | **Education level, N (%)** | |  | |  | |  | |  | |  | |
| Literacy | | 1,086 (53.11) | | 68 (27.20) | | 361 (42.62) | | 657 (69.30) | | <0.001 | |  |
| Illiteracy | | 959 (46.89) | | 182 (72.80) | | 486 (57.38) | | 291 (30.70) | |  |  |  |
|  | **Marital status, N (%)** | |  | |  | |  | |  | |  | |
| Married/separated | | 1,262 (61.71) | | 89 (35.60) | | 455 (53.72) | | 718 (75.74) | | <0.001 | |  |
| Divorced/widowed/never married | | 783 (38.29) | | 161 (64.40) | | 392 (46.28) | | 230 (24.26) | |  |  |  |
|  | **Self-rated economic status, N (%)** | |  | |  | |  | |  | |  | |
| Rich | | 264 (12.91) | | 29 (11.60) | | 101 (11.92) | | 134 (14.14) | | 0.225 | |  |
| Fair | | 1,467 (71.74) | | 178 (71.20) | | 604 (71.31) | | 685 (72.26) | |  |  |  |
| Poor | | 314 (15.35) | | 43 (17.20) | | 142 (16.77) | | 129 (13.61) | |  |  |  |
|  | **Smoking, N (%)** | |  | |  | |  | |  | |  | |
| Never | | 1,292 (63.18) | | 203 (81.20) | | 565 (66.71) | | 524 (55.27) | | <0.001 | |  |
| Ever/present | | 753 (36.82) | | 47 (18.80) | | 282 (33.29) | | 424 (44.73) | |  |  |  |
|  | **Alcohol assumption, N (%)** | |  | |  | |  | |  | |  | |
| Never | | 1,311 (64.11) | | 202 (80.80) | | 562 (66.35) | | 547 (57.70) | | <0.001 | |  |
| Ever/present | | 734 (35.89) | | 48 (19.20) | | 285 (33.65) | | 401 (42.30) | |  |  |  |
|  | **Physical activity/exercise, N (%)** | |  | |  | |  | |  | |  | |
| Never | | 1159 (56.67) | | 155 (62.00) | | 489 (57.73) | | 515 (54.32) | | 0.067 | |  |
| Ever/present | | 886 (43.43) | | 95 (38.00) | | 358 (42.27) | | 433 (45.68) | |  |  |  |
|  | **Self-rated health, N (%)** | |  | |  | |  | |  | |  | |
| Good | | 1,163 (56.87) | | 117 (46.80) | | 460 (54.31) | | 586 (61.81) | | <0.001 | |  |
| Fair | | 615 (30.07) | | 76 (30.40) | | 258 (30.46) | | 281 (29.64) | |  |  |  |
| Bad | | 267 (13.06) | | 57 (22.80) | | 129 (15.23) | | 81 (8.54) | |  |  |  |
|  | **Chronic diseases, N (%)** | |  | |  | |  | |  | |  | |
| 0 | | 888 (43.32) | | 88 (35.20) | | 351 (41.44) | | 449 (47.36) | | 0.001 | |  |
| 1 | | 654 (31.98) | | 87 (34.80) | | 268 (31.64) | | 299 (31.54) | |  |  |  |
| ≥2 | | 503 (24.60) | | 75 (30.00) | | 228 (26.92) | | 200 (21.10) | |  |  |  |
|  | IADL: Instrumental Activities of Daily Living | | | | | | | | | | | |

| Table S8 Fitting parameters for IADL GBTM according to the number of groups and trajectory shapes in older adults | | | | | | | | | | | |
| --- | --- | --- | --- | --- | --- | --- | --- | --- | --- | --- | --- |
| **Number of groups** | **Trajectory shapes^1^** | **BIC^2^** | **BIC^3^** | **AvePP** | | | | **OCC** | | | |
|  |  |  |  | **Group 1** | **Group 2** | **Group 3** | **Group 4** | **Group 1** | **Group 2** | **Group 3** | **Group 4** |
| 2 | 1 1 | -12378.21 | -12374.05 | 0.904 | 0.942 | \ | \ | 226.448 | 183.78 | \ | \ |
| 2 | 1 0 | -12576.92 | -12573.45 | 0.915 | 0.905 | \ | \ | 178.172 | 138.399 | \ | \ |
| 2 | 0 3 | -12643.43 | -12638.58 | 0.884 | 0.953 | \ | \ | 271.324 | 194.788 | \ | \ |
| 2 | 0 2 | -12643.03 | -12638.88 | 0.882 | 0.953 | \ | \ | 265.291 | 196.651 | \ | \ |
| 2 | 0 1 | -12652.65 | -12649.18 | 0.887 | 0.951 | \ | \ | 268.367 | 187.887 | \ | \ |
| 2 | 0 0 | -13044.7 | -13041.93 | 0.876 | 0.919 | \ | \ | 163.279 | 130.301 | \ | \ |
| 3 | 3 1 3 | -12261.93 | -12252.92 | 0.854 | 0.815 | 0.875 | \ | 363.656 | 83.181 | 119.659 | \ |
| 3 | 3 1 1 | -12269.76 | -12262.14 | 0.856 | 0.814 | 0.867 | \ | 345.889 | 79.880 | 117.908 | \ |
| 3 | 3 0 1 | -12351.56 | -12344.63 | 0.870 | 0.587 | 0.889 | \ | 181.549 | 113.196 | 100.875 | \ |
| 3 | 1 3 0 | -12273.73 | -12266.80 | 0.871 | 0.861 | 0.842 | \ | 284.336 | 86.586 | 163.856 | \ |
| 3 | 1 1 3 | -12266.50 | -12258.88 | 0.868 | 0.83 | 0.888 | \ | 540.105 | 95.386 | 123.218 | \ |
| 3 | 1 1 1 | -12275.46 | -12269.22 | 0.869 | 0.829 | 0.88 | \ | 518.940 | 91.254 | 118.410 | \ |
| 3 | 1 1 0 | -12302.87 | -12297.32 | 0.865 | 0.872 | 0.822 | \ | 241.574 | 94.106 | 161.026 | \ |
| 3 | 1 0 3 | -12367.40 | -12360.47 | 0.854 | 0.558 | 0.909 | \ | 160.743 | 141.014 | 118.975 | \ |
| 3 | 1 0 1 | -12375.73 | -12370.19 | 0.860 | 0.574 | 0.895 | \ | 168.797 | 111.737 | 106.433 | \ |
| 3 | 0 1 3 | -12367.4 | -12360.47 | 0.558 | 0.854 | 0.909 | \ | 141.015 | 160.743 | 118.975 | \ |
| 3 | 0 1 0 | -12434.61 | -12429.76 | 0.862 | 0.879 | 0.876 | \ | 651.938 | 105.609 | 141.569 | \ |
| 3 | 0 0 0 | -13017.6 | -13013.44 | 0.784 | 0.652 | 0.860 | \ | 207.092 | 51.178 | 83.236 | \ |
| 3 | 1 1 0 3 | -12239.17 | -12230.16 | 0.824 | 0.805 | 0.728 | 0.878 | 677.911 | 128.681 | 127.350 | 107.503 |
| 4 | 1 0 1 3 | -12271.35 | -12262.34 | 0.859 | 0.569 | 0.758 | 0.879 | 499.422 | 184.553 | 72.431 | 112.109 |
| 4 | 1 0 0 3^b^ | -12278.11 | -12269.80 | 0.826 | 0.554 | 0.828 | 0.841 | 224.679 | 238.496 | 144.129 | 78.488 |
| 4 | 0 3 1 3^b^ | -12256.38 | -12245.98 | 0.786 | 0.805 | 0.790 | 0.827 | 946.870 | 173.946 | 70.792 | 103.594 |
| 4 | 0 1 1 3 | -12271.35 | -12262.34 | 0.569 | 0.859 | 0.758 | 0.879 | 184.585 | 499.454 | 72.422 | 112.122 |
| 4 | 0 1 0 3^b^ | -12320.88 | -12312.56 | 0.816 | 0.827 | 0.572 | 0.881 | 906.784 | 120.291 | 144.024 | 99.588 |
| 4 | 3 1 1 1^b^ | 12257.04 | -12247.34 | 0.858 | 0.797 | 0.659 | 0.868 | 346.433 | 82.288 | 404.602 | 114.379 |
| 4 | 3 0 1 1^b^ | 12272.37 | -12263.36 | 0.846 | 0.787 | 0.860 | 0.739 | 176.109 | 888.387 | 95.735 | 103.032 |
| 4 | 1 1 1 1^b^ | 12264.61 | -12256.29 | 0.875 | 0.809 | 0.650 | 0.890 | 556.793 | 90.710 | 434.363 | 126.256 |
| 4 | 0 1 0 1^b^ | -12327.83 | -12320.90 | 0.822 | 0.814 | 0.610 | 0.867 | 924.815 | 108.666 | 123.687 | 93.362 |
| 4 | 0 0 1 1^b^ | 12327.83 | -12320.90 | 0.822 | 0.610 | 0.814 | 0.867 | 924.803 | 123.688 | 108.666 | 93.362 |
| 4 | 3 1 0 0 | -12284.32 | -12276.00 | 0.838 | 0.832 | 0.595 | 0.810 | 210.663 | 76.254 | 164.279 | 151.262 |
| 4 | 3 0 1 0 | -12284.32 | -12276.00 | 0.838 | 0.595 | 0.832 | 0.809 | 210.659 | 164.280 | 76.254 | 151.263 |
| 4 | 1 3 0 0^b^ | -12278.11 | -12269.80 | 0.826 | 0.841 | 0.554 | 0.828 | 224.686 | 78.493 | 238.478 | 144.123 |
| 4 | 1 1 3 0 | -12239.17 | -12230.16 | 0.824 | 0.805 | 0.878 | 0.728 | 677.915 | 128.678 | 107.493 | 127.352 |
| 4 | 1 1 0 0 | -12303.11 | -12296.18 | 0.829 | 0.832 | 0.567 | 0.810 | 204.072 | 75.919 | 160.633 | 143.833 |
| 4 | 1 0 3 0^b^ | -12278.11 | -12269.80 | 0.826 | 0.554 | 0.841 | 0.828 | 224.690 | 238.477 | 78.496 | 144.114 |
| 4 | 1 0 1 0 | -12303.11 | -12296.18 | 0.829 | 0.567 | 0.832 | 0.81 | 204.072 | 160.632 | 75.919 | 143.833 |
| 4 | 0 3 1 0 | -12284.32 | -12276.00 | 0.595 | 0.838 | 0.832 | 0.809 | 164.280 | 210.660 | 76.254 | 151.263 |
| 4 | 0 1 1 0 | -12303.11 | -12296.18 | 0.567 | 0.829 | 0.832 | 0.81 | 160.631 | 204.072 | 75.919 | 143.834 |
| ^1^Trajectory shapes; 0=zero-order; 1=linear; 2=quadratic; 3=cubic; ^2^BIC=Bayesian information criterion (for the total number of participants); ^3^BIC=Bayesian information criterion (for the total number of observations); AvePP: Average posterior probabilities; OCC: Odds of correct classification; ^a^The final model was chosen for trajectory analysis after a comprehensive comparison of BIC (BIC more approaching to 0), AvePP (Ideally, the average posterior probabilities (AvePP) were close to 1, at least higher than 0.7 for all groups), and OCC (greater than 5); ^b^One or more of the groups had a very small proportion of the observations (less than 5%). | | | | | | | | | | | |

| Table S9 Fitting parameters for BADL GBTM according to the number of groups and trajectory shapes in adults aged 60-79 years old | | | | | | | | | |
| --- | --- | --- | --- | --- | --- | --- | --- | --- | --- |
| **Number of groups** | **Trajectory shapes^1^** | **BIC^2^** | **BIC^3^** | **AvePP** | | | **OCC** | | |
|  |  |  |  | **Group 1** | **Group 2** | **Group 3** | **Group 1** | **Group 2** | **Group 3** |
| 2 | 3 1 | -1935.25 | -1929.71 | 0.846 | 0.953 | \ | 569.377 | 240.645 | \ |
| 2 | 3 0 | -1938.89 | -1934.04 | 0.863 | 0.901 | \ | 278.96 | 129.021 | \ |
| 2 | 1 1^a^ | -1930.89 | -1926.73 | 0.836 | 0.955 | \ | 555.218 | 248.959 | \ |
| 2 | 1 0 | -1935.29 | -1931.82 | 0.892 | 0.892 | \ | 371.052 | 116.741 | \ |
| 2 | 0 1^b^ | -1967.24 | -1963.78 | 0.806 | 0.984 | \ | 1477.62 | 665.583 | \ |
| 2 | 0 0 | -2063.9 | -2061.13 | 0.795 | 0.926 | \ | 362.761 | 148.857 | \ |
| 3 | 1 1 2 | -1940.34 | -1933.41 | 0.790 | 0.869 | 0.811 | 175.82 | 1078.726 | 66.159 |
| ^1^Trajectory shapes; 0=zero-order; 1=linear; 2=quadratic; 3=cubic; ^2^BIC=Bayesian information criterion (for the total number of participants); ^3^BIC=Bayesian information criterion (for the total number of observations); AvePP: Average posterior probabilities; OCC: Odds of correct classification; ^a^The final model was chosen for trajectory analysis after a comprehensive comparison of BIC (BIC more approaching to 0), AvePP (Ideally, the average posterior probabilities (AvePP) were close to 1, at least higher than 0.7 for all groups), and OCC (greater than 5); ^b^One or more of the groups had a very small proportion of the observations (less than 5%). | | | | | | | | | |

| Table S10 Fitting parameters for BADL GBTM according to the number of groups and trajectory shapes in adults aged 80+ years old | | | | | | | | | |
| --- | --- | --- | --- | --- | --- | --- | --- | --- | --- |
| **Number of groups** | **Trajectory shapes^1^** | **BIC^2^** | **BIC^3^** | **AvePP** | | | **OCC** | | |
|  |  |  |  | **Group 1** | **Group 2** | **Group 3** | **Group 1** | **Group 2** | **Group 3** |
| 2 | 3 0 | -1669.66 | -1664.81 | 0.956 | 0.833 | \ | 1144.597 | 333.464 | \ |
| 2 | 0 3^b^ | -1703.82 | -1698.97 | 0.795 | 0.975 | \ | 2683.128 | 1166.008 | \ |
| 2 | 0 1 | -1701.83 | -1698.36 | 0.778 | 0.971 | \ | 2030.359 | 1025.602 | \ |
| 2 | 0 0 | -1852.79 | -1850.02 | 0.912 | 0.66 | \ | 480.949 | 153.361 | \ |
| 3 | 1 1 0^a^ | -1668.73 | -1663.18 | 0.901 | 0.706 | 0.814 | 978.066 | 283.216 | 271.014 |
| ^1^Trajectory shapes; 0=zero-order; 1=linear; 2=quadratic; 3=cubic; ^2^BIC=Bayesian information criterion (for the total number of participants); ^3^BIC=Bayesian information criterion (for the total number of observations); AvePP: Average posterior probabilities; OCC: Odds of correct classification; ^a^The final model was chosen for trajectory analysis after a comprehensive comparison of BIC (BIC more approaching to 0), AvePP (Ideally, the average posterior probabilities (AvePP) were close to 1, at least higher than 0.7 for all groups), and OCC (greater than 5); ^b^One or more of the groups had a very small proportion of the observations (less than 5%). | | | | | | | | | |

# Fig S1 BADL trajectories in older adults by age groups

**Fig S1a BADL trajectories in adults aged 60-79 years old Fig S1b BADL trajectories in adults aged 80+ years old**

| Table S11 Fitting parameters for IADL GBTM according to the number of groups and trajectory shapes in adults aged 60-79 years old | | | | | | | | | |
| --- | --- | --- | --- | --- | --- | --- | --- | --- | --- |
| **Number of groups** | **Trajectory shapes^1^** | **BIC^2^** | **BIC^3^** | AvePP | | | OCC | | |
|  |  |  |  | Group 1 | Group 2 | Group 3 | Group 1 | Group 2 | Group 3 |
| 2 | 2 2 | -7640.38 | -7634.84 | 0.876 | 0.922 | \ | 247.623 | 184.783 | \ |
| 2 | 2 0 | -7722.46 | -7718.3 | 0.901 | 0.872 | \ | 189.305 | 154.665 | \ |
| 2 | 1 3 | -7645.89 | -7640.34 | 0.858 | 0.925 | \ | 214.279 | 192.15 | \ |
| 2 | 1 1 | -7648.53 | -7644.37 | 0.86 | 0.924 | \ | 215.777 | 190.807 | \ |
| 2 | 1 0 | -7767.36 | -7763.90 | 0.876 | 0.847 | \ | 161.869 | 114.594 | \ |
| 2 | 0 3 | -7805.76 | -7800.91 | 0.843 | 0.953 | \ | 398.265 | 251.171 | \ |
| 2 | 0 2 | -7805.62 | -7801.46 | 0.853 | 0.951 | \ | 430.107 | 240.905 | \ |
| 2 | 0 1 | -7805.13 | -7801.67 | 0.848 | 0.951 | \ | 405.526 | 241.840 | \ |
| 2 | 0 0 | -8097.81 | -8095.04 | 0.855 | 0.872 | \ | 188.458 | 111.345 | \ |
| 3 | 1 3 0 | -7615.02 | -7608.09 | 0.843 | 0.885 | 0.762 | 373.651 | 138.446 | 146.13 |
| 3 | 1 1 3 | -7618.11 | -7610.48 | 0.801 | 0.801 | 0.87 | 632.285 | 104.904 | 143.742 |
| 3 | 1 1 1 | -7620.83 | -7614.59 | 0.805 | 0.802 | 0.856 | 601.673 | 102.860 | 131.139 |
| 3 | 1 1 0 | -7622.79 | -7617.25 | 0.881 | 0.846 | 0.735 | 134.644 | 360.834 | 132.706 |
| 3 | 1 0 0 | -7684.50 | -7679.65 | 0.849 | 0.849 | 0.88 | 107.235 | 1648.058 | 203.701 |
| 3 | 0 2 0^b^ | -7673.74 | -7668.2 | 0.841 | 0.893 | 0.841 | 1854.562 | 163.245 | 139.087 |
| 3 | 0 1 3^b^ | -7654.07 | -7647.14 | 0.470 | 0.841 | 0.906 | 368.416 | 193.860 | 153.383 |
| 3 | 0 1 0^b^ | -7684.5 | -7679.65 | 0.849 | 0.849 | 0.88 | 1648.048 | 107.237 | 203.701 |
| ^1^Trajectory shapes; 0=zero-order; 1=linear; 2=quadratic; 3=cubic; ^2^BIC=Bayesian information criterion (for the total number of participants); ^3^BIC=Bayesian information criterion (for the total number of observations); AvePP: Average posterior probabilities; OCC: Odds of correct classification; ^a^The final model was chosen for trajectory analysis after a comprehensive comparison of BIC (BIC more approaching to 0), AvePP (Ideally, the average posterior probabilities (AvePP) were close to 1, at least higher than 0.7 for all groups), and OCC (greater than 5); ^b^One or more of the groups had a very small proportion of the observations (less than 5%). | | | | | | | | | |
| **Table S12 Fitting parameters for IADL GBTM according to the number of groups and trajectory shapes in adults aged 80+ years old** | | | | | | | | | |

| **Number of groups** | **Trajectory shapes^1^** | **BIC^2^** | **BIC^3^** | AvePP | | | | OCC | | | |
| --- | --- | --- | --- | --- | --- | --- | --- | --- | --- | --- | --- |
|  |  |  |  | Group 1 | Group 2 | Group 3 | Group 4 | Group 1 | Group 2 | Group 3 | Group 4 |
| 2 | 1 2 | -4412.22 | -4407.37 | 0.886 | 0.915 | \ | \ | 535.411 | 548.189 | \ | \ |
| 2 | 1 1 | -4430.94 | -4426.78 | 0.883 | 0.907 | \ | \ | 496.692 | 515.969 | \ | \ |
| 2 | 1 0 | -4481.86 | -4478.39 | 0.92 | 0.887 | \ | \ | 556.794 | 587.416 | \ | \ |
| 2 | 0 1 | -4520.88 | -4517.41 | 0.844 | 0.946 | \ | \ | 743.351 | 651.306 | \ | \ |
| 2 | 0 0 | -4674.12 | -4671.35 | 0.846 | 0.871 | \ | \ | 365.306 | 354.850 | \ | \ |
| 3 | 1 2 0 | -4400.5 | -4394.26 | 0.868 | 0.846 | 0.781 | \ | 741.457 | 287.104 | 607.429 | \ |
| 3 | 1 1 2 | -4401.17 | -4394.24 | 0.815 | 0.804 | 0.878 | \ | 838.489 | 273.262 | 529.259 | \ |
| 3 | 1 1 1 | -4417.49 | -4411.25 | 0.820 | 0.813 | 0.845 | \ | 795.057 | 270.962 | 447.984 | \ |
| 3 | 1 1 0 | -4425.23 | -4419.68 | 0.869 | 0.827 | 0.778 | \ | 667.655 | 250.235 | 711.793 | \ |
| 3 | 1 0 2 | -4418.22 | -4411.98 | 0.842 | 0.571 | 0.875 | \ | 394.855 | 568.704 | 383.358 | \ |
| 3 | 1 0 1 | -4435.12 | -4429.58 | 0.846 | 0.586 | 0.844 | \ | 377.059 | 357.136 | 352.929 | \ |
| 4 | 1 0 0 1 | -4418.66 | -4411.04 | 0.854 | 0.573 | 0.786 | 0.770 | 671.813 | 471.951 | 655.942 | 200.603 |
| 4 | 1 0 1 1 | -4418.38 | -4410.75 | 0.834 | 0.636 | 0.738 | 0.812 | 808.109 | 376.013 | 232.549 | 398.168 |
| 4 | 1 0 0 1 | -4429.29 | -4422.36 | 0.854 | 0.573 | 0.786 | 0.77 | 671.813 | 471.951 | 655.942 | 200.603 |
| 4 | 0 1 1 1 | -4418.38 | -4410.75 | 0.636 | 0.834 | 0.738 | 0.812 | 376.011 | 808.097 | 232.550 | 398.173 |
| 4 | 1 1 0 0 | -4429.29 | -4422.36 | 0.854 | 0.770 | 0.573 | 0.786 | 671.788 | 200.603 | 471.959 | 655.968 |
| 4 | 1 0 1 0 | -4429.29 | -4422.36 | 0.854 | 0.573 | 0.770 | 0.786 | 671.786 | 471.961 | 200.604 | 655.964 |
| 4 | 0 1 1 0 | -4429.29 | -4422.36 | 0.573 | 0.854 | 0.770 | 0.786 | 471.955 | 671.802 | 200.602 | 655.956 |
| ^1^Trajectory shapes; 0=zero-order; 1=linear; 2=quadratic; 3=cubic; ^2^BIC=Bayesian information criterion (for the total number of participants); ^3^BIC=Bayesian information criterion (for the total number of observations); AvePP: Average posterior probabilities; OCC: Odds of correct classification; ^a^The final model was chosen for trajectory analysis after a comprehensive comparison of BIC (BIC more approaching to 0), AvePP (Ideally, the average posterior probabilities (AvePP) were close to 1, at least higher than 0.7 for all groups), and OCC (greater than 5); ^b^One or more of the groups had a very small proportion of the observations (less than 5%). | | | | | | | | | | | |

# Fig S2 IADL trajectories in older adults by age groups

**Fig S2a IADL trajectories in adults aged 60-79 years old Fig S2b IADL trajectories in adults aged 80+ years old**

|  | Table S13 Association of IC with trajectories of BADL and IADL in adults aged 60-79 years old | | | | | | | |
| --- | --- | --- | --- | --- | --- | --- | --- | --- |
|  | **Characteristics** | | BADL **trajectories^a^** | | IADL **trajectories^b^** | | | |
|  |  |  | **Group 1  (***High and declining***),  OR (95% CI)^c^** | | **Group 1 (***Medium and severely declining***),  OR (95% CI)^d^** | | **Group 2  (***High and moderately declining***),  OR (95% CI)^d^** | |
|  | **IC deficit score** | | 1.22 (1.00,1.48)^*^ | | 1.76 (1.45, 2.14)^***^ | | 1.20 (1.04, 1.39)^*^ | |
|  | **Sex** | |  | |  | |  | |
| Male | | reference | | reference | | reference | |  |
| Female | | 1.88 (1.03, 3.43)^*^ | | 3.60 (2.10, 6.17)^***^ | | 1.98 (1.40, 2.79)^***^ | |  |
|  | **Ethnicity** | |  | |  | |  | |
| Han | | reference | | reference | | reference | |  |
| Minority | | 0.53 (0.19, 1.50) | | 0.49 (0.23, 1.05) | | 0.54 (0.33, 0.88)^*^ | |  |
|  | **Educational level** | |  | |  | |  | |
| Literacy | | reference | | reference | | reference | |  |
| Illiteracy | | 1.65 (1.07, 2.56)^*^ | | 2.88 (1.92, 4.32)^***^ | | 1.78 (1.33, 2.38)^***^ | |  |
|  | **Marital status** | |  | |  | |  | |
| Married/separated | | reference | | reference | | reference | |  |
| Divorced/widowed/never married | | 0.96 (0.62, 1.49) | | 1.79 (1.18, 2.71)^**^ | | 1.52 (1.11, 1.07)^**^ | |  |
|  | **Self-rated economic status** | |  | |  | |  | |
| Rich | | reference | | reference | | reference | |  |
| Fair | | 0.72 (0.40, 1.31) | | 0.90 (0.50, 1.59) | | 1.20 (0.83, 1.74) | |  |
| Poor | | 0.80 (0.38, 1.68) | | 1.32 (0.64, 2.70) | | 1.55 (0.94, 2.57) | |  |
|  | **Smoking status** | |  | |  | |  | |
| Never | | reference | | reference | | reference | |  |
| Ever/present | | 1.29 (0.73, 2.28) | | 1.01 (0.60, 1.71) | | 1.26 (0.91, 1.74) | |  |
|  | **Alcohol consumption status** | |  | |  | |  | |
| Never | | reference | | reference | | reference | |  |
| Ever/present | | 1.17 (0.71, 1.94) | | 1.26 (0.80, 2.00) | | 1.05 (0.79, 1.41) | |  |
|  | **Physical activity/exercise status** | |  | |  | |  | |
| Yes | | reference | | reference | | reference | |  |
| No | | 1.03 (0.68, 1.57) | | 0.82 (0.56, 1.21) | | 1.07 (0.82, 1.38) | |  |
|  | **Self-rated health** | |  | |  | |  | |
| Good | | reference | | reference | | reference | |  |
| Fair | | 0.91 (0.56, 1.48) | | 1.20 (0.78, 1.83) | | 1.02 (0.77, 1.36) | |  |
| Bad | | 1.77 (1.02, 3.08)^*^ | | 3.02 (1.69, 5.40 )^***^ | | 1.40 (0.87, 2.24) | |  |
|  | **Chronic diseases** | |  | |  | |  | |
| 0 | | reference | | reference | | reference | |  |
| 1 | | 0.76 (0.45, 1.28) | | 1.84 (1.17, 2.88)^**^ | | 1.69 (1.27, 2.26)^***^ | |  |
| ≥2 | | 1.32 (0.80, 2.16) | | 3.00 (1.86, 4.84)^***^ | | 2.11 (1.50, 2.97)^***^ | |  |
|  | BADL: Basic Activities of Daily Living; IADL: Instrumental Activities of Daily Living; ^a^: Group 2 (High and stable group) as the reference; ^b^: Group 3 (High and slightly declining group) as the reference; ^c^: Evaluated by a generalized linear model with binomial family and default logit link; ^d^: Evaluated by a multinomial logistic regression model. ^***^: *P*<0.001; ^**^: *P*<0.01； ^*^: *P*<0.05 | | | | | | | |

|  | Table S14 Association of IC with trajectories of BADL and IADL in adults aged 80+ years old | | | | | | | |
| --- | --- | --- | --- | --- | --- | --- | --- | --- |
|  | **Characteristics** | | **BADL Trajectories^a^** | | **IADL trajectories^b^** | | | |
|  |  |  | **Group 1 (***High and declining***),  OR (95% CI)^c^** | | **Group 1 (***Low and declining***),  OR (95% CI)^d^** | | **Group 2  (***Medium and severely declining***),  OR (95% CI)^d^** | |
|  | **IC deficit score** | | 1.16 (1.01,1.33)^*^ | | 1.90 (1.52, 2.38)^***^ | | 1.23 (1.04, 1.45)^*^ | |
|  | **Sex** | |  | |  | |  | |
| Male | | reference | | reference | | reference | |  |
| Female | | 1.21 (0.76, 1.92) | | 4.95 (2.17, 11.30)^***^ | | 2.49 (1.51, 4.11)^***^ | |  |
|  | **Ethnicity** | |  | |  | |  | |
|  | Han | | reference | | reference | | reference | |
|  | Minority | | 0.64 (0.32, 1.30) | | 1.14 (0.41, 3.19) | | 0.54 (0.25, 1.19) | |
|  | **Educational level** | |  | |  | |  | |
| Literacy | | reference | | reference | | reference | |  |
| Illiteracy | | 0.87 (0.57, 1.31) | | 0.88 (0.45, 1.75) | | 1.31 (0.84, 2.06) | |  |
|  | **Marital status** | |  | |  | |  | |
| Married/separated | | reference | | reference | | reference | |  |
| Divorced/widowed/never married | | 1.02 (0.69, 1.49) | | 1.16 (0.60, 2.25) | | 1.09 (0.72, 1.66) | |  |
|  | **Self-rated economic status** | |  | |  | |  | |
| Rich | | reference | | reference | | reference | |  |
| Fair | | 1.17 (0.70, 1.94) | | 0.77 (0.34, 1.74) | | 1.05 (0.59, 1.85) | |  |
| Poor | | 0.80 (0.41, 1.55) | | 0.43 (0.14, 1.29) | | 0.86 (0.41, 1.81) | |  |
|  | **Smoking status** | |  | |  | |  | |
| Never | | reference | | reference | | reference | |  |
| Ever/present | | 1.28 (0.80, 2.04) | | 2.14 (0.96, 4.80) | | 1.02 (0.61, 1.70) | |  |
|  | **Alcohol consumption status** | |  | |  | |  | |
| Never | | reference | | reference | | reference | |  |
| Ever/present | | 0.60 (0.39, 0.92)^*^ | | 0.36 (0.16, 0.80)^*^ | | 0.87 (0.55, 1.38) | |  |
|  | **Physical activity/exercise status** | |  | |  | |  | |
| Yes | | reference | | reference | | reference | |  |
| No | | 1.15 (0.79, 1.66) | | 1.24 (0.67,2.29) | | 1.06 (0.70, 1.60) | |  |
|  | **Self-rated health** | |  | |  | |  | |
| Good | | reference | | reference | | reference | |  |
| Fair | | 0.79 (0.53, 1.17) | | 1.15 (0.60, 2.23) | | 1.07 (0.69, 1.67) | |  |
| Bad | | 0.74 (0.42, 1.31) | | 2.25 (0.93, 5.45) | | 1.40 (0.72, 2.72) | |  |
|  | **Chronic diseases** | |  | |  | |  | |
| 0 | | reference | | reference | | reference | |  |
| 1 | | 1.27 (0.86, 1.89) | | 1.34 (0.68, 2.63) | | 1.54 (0.99, 2.39) | |  |
| ≥2 | | 1.72 (1.06, 2.79)^*^ | | 2.83 (1.31, 6.10)^**^ | | 1.68 (0.97, 2.93) | |  |
|  | BADL: Basic Activities of Daily Living; IADL: Instrumental Activities of Daily Living; ^a^: Group 2 (High and stable group) as the reference; ^b^: Group 3 (High and slightly declining group) as the reference; ^c^: Evaluated by a generalized linear model with binomial family and default logit link; ^d^: Evaluated by a multinomial logistic regression model. ^***^: *P*<0.001; ^**^: *P*<0.01； ^*^: *P*<0.05. | | | | | | | |

| Table S15 Longitudinal associations between IC and the annual rate of change in physical function in adults aged 60-79 years old | | | |
| --- | --- | --- | --- |
| Physical function measures^a^ | Model terms | Adjusted coefficient (95% CI)^b^ | *P* |
| BADL | IC deficit score | -0.015 (-0.026, -0.004) | 0.01 |
|  | Time | -0.019 (-0.026, -0.012) | <0.001 |
|  | IC×Time | -0.002 (-0.006, 0.002) | 0.320 |
| IADL | IC deficit score | -0.197 (-0.262, -0.132) | <0.001 |
|  | Time | -0.149 (-0.172, -0.125) | <0.001 |
|  | IC×Time | -0.022 (-0.036, 0.007) | 0.003 |
| IC: Intrinsic capacity; CI: Confidence Interval; BADL: Basic activities of daily living; IADL: Instrumental activities of daily living.  ^a^: Higher scores indicate better physical function. ^b^: Adjusted for age, sex, ethnicity, education, marital status, self-rated economic status, smoking status, alcohol consumption status, exercise status, self-rated health, and number of chronic diseases. | | | |

| Table S16 Longitudinal associations between IC and the annual rate of change in physical function in adults aged 80+ years old | | | |
| --- | --- | --- | --- |
| Physical function measures^a^ | Model terms | Adjusted coefficient (95% CI)^b^ | *P* |
| BADL | IC deficit score | -0.045 (-0.070, -0.021) | <0.001 |
|  | Time | -0.066 (-0.086, -0.045) | <0.001 |
|  | IC×Time | -0.001 (-0.009, 0.008) | 0.203 |
| IADL | IC deficit score | -0.611 (-0.740, -0.482) | <0.001 |
|  | Time | -0.397 (-0.448, -0.347) | <0.001 |
|  | IC×Time | -0.044 (-0.023, -0.065) | <0.001 |
| IC: Intrinsic capacity; CI: Confidence Interval; BADL: Basic activities of daily living; IADL: Instrumental activities of daily living.  ^a^: Higher scores indicate better physical function. ^b^: Adjusted for age, sex, ethnicity, education, marital status, self-rated economic status, smoking status, alcohol consumption status, exercise status, self-rated health, and number of chronic diseases. | | | |

| **Table S17 Panel A. All-available trajectory analyses** | | | | |
| --- | --- | --- | --- | --- |
| **Outcome / estimate** | **Primary complete-case analysis** | *P* | **All-available sensitivity analysis** | *P* |
| BADL | n=2,045 |  | n=7,769 |  |
| High and declining trajectory | 18.1% |  | **28.1%** |  |
| High and stable trajectory | 81.9% |  | **71.9%** |  |
| IC deficit score: declining vs stable | OR **1.22 (1.09–1.37)** | 0.001 | OR **1.34 (1.28–1.40)** | <0.001 |
| IADL | n=2,045 |  | n=7,825 | <0.001 |
| Severe-decline trajectory | 13.0% |  | **25.3%** | <0.001 |
| Moderate-decline trajectory | 41.3% |  | **39.7%** | <0.001 |
| Mild-decline trajectory | 45.7% |  | **35.0%** |  |
| IC deficit score: severe vs mild | RRR **1.94 (1.66–2.27)** | <0.001 | RRR **2.13 (1.99–2.29)** | <0.001 |
| IC deficit score: moderate vs mild | RRR **1.37 (1.22–1.53)** | <0.001 | RRR **1.40 (1.32–1.48)** | <0.001 |

| **Table S17 Panel B. MAR and MNAR sensitivity analyses for the IC deficit score × time interaction** | | | | | |
| --- | --- | --- | --- | --- | --- |
| **Missing-data assumption** | **Participants** | **BADL IC×time β (95% CI)** | **P** | **IADL IC×time β (95% CI)** | *P* |
| Primary complete-case analysis | 2,045 | -0.0070 (−0.0100，-0.0030) | 0.001 | -0.0090 (−0.0210 ,0.0020) | 0.111 |
| MAR multiple imputation | 14,658 | -**0.0121 (−0.0190,** -**0.0051)** | **0.001** | **0.0436 (0.0334 , 0.0539)** | **<0.001** |
| MNAR, δ=−0.5 | 14,658 | **-0.0127 (−0.0192, -0.0062)** | **<0.001** | **0.0467 (0.0373 , 0.0561)** | **<0.001** |
| MNAR, δ=−1.0 | 14,658 | **-0.0134 (−0.0195 , -0.0073)** | **<0.001** | **0.0498 (0.0413 , 0.0582)** | **<0.001** |

| Table S18 Sensitivity analysis retaining MMSE and BMI as continuous measures | | | | | | | | | | |
| --- | --- | --- | --- | --- | --- | --- | --- | --- | --- | --- |
| Outcome | Baseline measure × time | | β | | 95% CI | | *P* | | | |
| BADL | Continuous MMSE × time | | 0.0035 | | 0.0023 to 0.0047 | | <0.001 | | | |
|  | Continuous BMI terms × time | | — | | — | | 0.250ᵃ | | | |
|  | Other four IC deficits × time | | -0.0130 | | -0.0192 to -0.0069 | | <0.001 | | | |
| IADL | Continuous MMSE × time | | 0.0014 | | -0.0014 to 0.0041 | | 0.333 | | | |
|  | Continuous BMI terms × time | | — | | — | | 0.288ᵃ | | | |
|  | Other four IC deficits × time | | -0.0085 | | -0.0223 to 0.0052 | | 0.224 | | | |
| BADL, basic activities of daily living; IADL, instrumental activities of daily living; IC, intrinsic capacity; MMSE, Mini-Mental State Examination; BMI, body mass index; CI, confidence interval.  Sensitivity analyses included 2,027 participants with complete information on baseline MMSE, BMI, the remaining four IC domains, covariates, and BADL/IADL measurements at all four survey waves. MMSE was modeled continuously and centered at 24 points. BMI was modeled continuously using linear and quadratic terms and centered at 21.7 kg/m² to allow for a potentially nonlinear association. The remaining four-domain IC deficit score was the sum of locomotor, visual, hearing, and psychological deficits. Models included interactions of these baseline measures with time and were adjusted for the same baseline covariates as the primary mixed-effects analysis, with a participant-specific random intercept.  **ᵃ P value from a joint Wald test of the linear BMI×time and quadratic BMI²×time interaction terms.** | | | | | | | | | | |
| Table S19 Baseline characteristics of participants included and not included in the primary complete-case analysis | | | | | | | |  |  |  |
| **Baseline Characteristics** | | **Not included (n=12,613)** | | **Included (n=2,045)** | | ***P*** | |  |  |  |
| **IC deficit score, mean (SD)** | | **2.55 (1.60)** | | **1.50 (1.13)** | | **<0.001** | |  |  |  |
| **BADL score, mean (SD)** | | **5.49 (1.28)** | | **5.97 (0.28)** | | **<0.001** | |  |  |  |
| **IADL score, mean (SD)** | | **4.43 (3.13)** | | **7.14 (1.72)** | | **<0.001** | |  |  |  |
| **Age (years old), N (%)** | |  | |  | | **<0.001** | |  |  |  |
| 60-79 | | 3,007 (23.84) | | 1,481 (72.42) | |  | |  |  |  |
| ≥80 | | 9,606 (76.16) | | 564 (27.58) | |  |  |  |  |  |
| **Gender, N (%)** | |  | |  | | **0.007** | |  |  |  |
| Male | | 5,600 (44.40) | | 973 (47.58) | |  | |  |  |  |
| Female | | 7,013 (55.60) | | 1,072 (52.42) | |  |  |  |  |  |
| **Ethnicity, N (%)** | |  | |  | | **0.782** | |  |  |  |
| Han | | 11,813 (93.66) | | 1,912 (93.50) | |  | |  |  |  |
| Minority | | 800 (6.34) | | 133 (6.50) | |  |  |  |  |  |
| **Education level, N (%)** | |  | |  | | **<0.001** | |  |  |  |
| Literacy | | 4,811 (38.14) | | 1,086 (53.11) | |  | |  |  |  |
| Illiteracy | | 7,802 (61.86) | | 959 (46.89) | |  |  |  |  |  |
| **Marital status, N (%)** | |  | |  | | <0.001 | |  |  |  |
| Married/separated | | 3,932 (31.17) | | 1,262 (61.71) | |  | |  |  |  |
| Divorced/widowed/never married | | 8,681 (68.83) | | 783 (38.29) | |  | |  |  |  |
| **Self-rated economic status, N (%)** | |  | |  | | 0.012 | |  |  |  |
| Rich | | 1,735 (13.76) | | 264 (12.91) | |  | |  |  |  |
| Fair | | 8,652 (68.60) | | 1,467 (71.74) | |  | |  |  |  |
| Poor | | 2,226 (17.65) | | 314 (15.35) | |  | |  |  |  |
| **Smoking, N (%)** | |  | |  | | <0.001 | |  |  |  |
| Never | | 10,409 (82.53) | | 1,573 (76.92) | |  | |  |  |  |
| Ever/present | | 2,204 (17.47) | | 472 (23.08) | |  | |  |  |  |
| **Alcohol assumption, N (%)** | |  | |  | | <0.001 | |  |  |  |
| Never | | 8,722 (69.15) | | 1,311 (64.11) | |  | |  |  |  |
| Ever/present | | 3,891 (30.85) | | 734 (35.89) | |  | |  |  |  |
| **Physical activity/exercise, N (%)** | |  | |  | | 0.192 | |  | |  |
| Never | | 7,342 (58.21) | | 1,159 (56.67) | |  | |  |  |  |
| Ever/present | | 5,271 (41.79) | | 886 (43.33) | |  | |  |  |  |
| **Self-rated health, N (%)** | |  | |  | | <0.001 | | | |  |
| Good | | 6,057 (48.02) | | 1,163 (56.87) | |  | |  |  |  |
| Fair | | 4,438 (35.19) | | 615 (30.07) | |  | |  |  |  |
| Bad | | 2,118 (16.79) | | 267 (13.06) | |  | |  |  |  |
| **Chronic diseases, N (%)** | |  | |  | | 0.460 | | | |  |
| 0 | | 5,415 (42.93) | | 888 (43.42) | |  | |  |  |  |
| 1 | | 3,935 (31.20) | | 654 (31.98) | |  | |  |  |  |
| ≥2 | | 3,263 (25.87) | | 503 (24.60) | |  | | | |  |
| IC, intrinsic capacity; BADL, basic activities of daily living; IADL, instrumental activities of daily living; SD, standard deviation. Values are presented as mean (SD) for continuous variables and n (%) for categorical variables. The included group comprised baseline-eligible participants with complete BADL and IADL measurements at all four survey waves (2008, 2011, 2014, and 2018). The not-included group comprised baseline-eligible participants who did not meet these complete follow-up criteria.   P-values were calculated using Welch’s two-sample t test for continuous variables and Pearson’s chi-square test for categorical variables. P values <0.001 are reported as P<0.001. | | | | | | | | |  |  |
